# Zebrafish macroH2A variants have distinct embryo localization and function

**DOI:** 10.1038/s41598-019-45058-6

**Published:** 2019-06-14

**Authors:** E. Gonzalez-Munoz, Y. Arboleda-Estudillo, S. K. Chanumolu, H. H. Otu, J. B. Cibelli

**Affiliations:** 1 Andalusian Laboratory of Cell Reprogramming (LARCel), Andalusian Center for Nanomedicine and Biotechnology-BIONAND, 29590 Málaga, Spain; 2 Department of Cell Biology, Genetics and Physiology, University of Málaga, 29071 Málaga, Spain; 3 Networking Research Center on Bioengineering, Biomaterials and Nanomedicine, (CIBER-BBN), 29071 Málaga, Spain; 4 Department of Electrical and Computer Engineering, University of Nebraska-Lincoln, Lincoln, NE, 68588 USA; 5 Department of Animal Science and Large Animal Clinical Sciences Department, Michigan State University, East Lansing, MI, 48824 USA

**Keywords:** Epigenetics, Zebrafish

## Abstract

Mouse and cell-based studies have shown that macroH2A histone variants predominantly associate with heterochromatin. Functional studies found that macroH2As are involved in gene repression, inhibiting the acquisition of pluripotency and preserving cell differentiation. However, only a few studies have analysed the role of macroH2A during early embryo development. We report the development of transgenic zebrafish lines expressing macroH2A isoforms (mH2A1 and mH2A2) fusion proteins (with GFP) under identified endogenous promoters. We found that mH2A1 and mH2A2 have different spatial and temporal expression patterns during embryonic development. mH2A1 is expressed mostly in the extraembryonic Yolk Syncytial Layer (YSL) starting before shield stage and decreasing once morphogenesis is completed. mH2A2 expression lags behind mH2A1, becoming evident at 24 hpf, within the whole body of the embryo proper. Our ChIP-seq analysis showed that mH2A1 and mH2A2 bind to different DNA regions, changing dramatically after gastrulation. We further analysed RNA-seq data and showed that there is not a general/unspecific repressing function of mH2A1 or mH2A2 associated with heterochromatin but a fine regulation depending on cell types and stage of development. mH2A1 downregulates DNA expression in specific cells and embryo stages and its effect is independent of heterochromatin formation but it is correlated with nucleus quiescence instead. Whereas mH2A2 DNA association correlates with upregulation of differentially expressed genes between 75% epiboly and 24 hpf stages. Our data provide information for underlying molecules that participate in crucial early developmental events, and open new venues to explore mH2A related mechanisms that involve cell proliferation, differentiation, migration and metabolism.

## Introduction

Canonical histone exchange is one of the most notable epigenetic alterations that occur in the nucleosome during oocyte fertilization, embryo development and nuclear reprogramming. Among all histone variants, macroH2A (mH2A) differs significantly from H2A, its canonical counterpart. It possesses an additional extranucleosomal C-terminal macrodomain that protrudes out, altering the nucleosome structure^1^. Although generally referred to as macroH2A (mH2A), two different genes encode for two isoforms in vertebrates: *mH2A1* and *mH2A2*. In mammals and other higher vertebrates, *mH2A1* can be further alternatively spliced, with cells containing three different mH2A proteins that are distinguished as mH2A1.1, m H2A1.2 and mH2A2^2–4^.

mH2A was first associated with heterochromatinization and gene repression as it was described to be associated with the inactive X chromosome and centrosomes^5^. Biochemical studies show that the structure of nucleosomes containing mH2A is different and it correlates with the inaccessibility of DNA for certain transcription factors suggesting that mH2A could be an epigenetic marker for gene silencing^6,7^.

mH2A isoforms have been studied in mouse development and mouse embryonic stem cells (mESCs)^8–11^. Recent studies show that after mouse fertilization, mH2A is evicted from the maternal genome, gradually reappearing with the start of lineage specification, at the onset of mouse embryonic differentiation *in vivo*^9,12,13^, leading to the notion that deposition of mH2A is globally enriched in differentiated cells compared to their pluripotent counterparts^11,14^ and that they constitute a barrier for cell reprogramming, in mouse^9,11,15^ and human somatic cells^16^. However the role of these isoforms on self-renewal seems to be dispensable; there are some conflicting results as to whether they are necessary for ESCs proper differentiation or not, with evidence supporting both hypotheses^10,11,17^.

The differential participation of both mH2A isoforms during pluripotency acquisition was analysed using genetically modified mouse models deficient for each, or both isoforms. The results suggested that mH2A1 and mH2A2 act cooperatively. There is some disagreement about the specific role of each isoform during cell reprogramming, with some reports stating a major role of mH2A2^9,18^ whereas others mH2A1^16^. It appears that the two isoforms have redundant functions during mouse embryo development, as double knock-out (dKO) mice are viable and free of obvious developmental defects^11^.

Most of the data for mH2A isoforms function and localization come from late embryonic stages, pluripotent cells or adult somatic cells. Little is known about their role during early embryo development.

Zebrafish (zf) is an ideal organism for *in vivo* study of early embryo development. Both mH2A homologous genes, mH2A1 and mH2A2, can be found in the zebrafish genome. These proteins are 70% identical to their human counterparts^19^. Zebrafish mH2A1 genomic DNA is annotated to encode both mutual exclusive 5′UTR non-coding exons (h2afy-201 and h2afy-202 transcripts)^20^, that yield the same 357aa mH2A1 protein, that shows functional and protein sequence conservation to human and mouse mH2A1.1 splice variant^21,22^. To the best of our knowledge macroH2A1 splicing variants affecting coding regions, and thus yielding different (mH2A1.1 and mH2A1.2) variants have not been annotated so far.

Here, we report that during zebrafish embryogenesis, mH2A isoforms have different temporal and spatial expression patterns and target different DNA regions of the genome. Specific cells expressing either mH2A1 or mH2A2 have different global RNA expression profiles, suggesting that these proteins have different, but highly regulated developmental roles during embryogenesis. The most significant change in DNA targeting was observed after gastrulation, suggesting a key role at the onset of somatic cell differentiation. We found that mH2A1 expression is almost exclusively restricted to the extraembryonic yolk syncytial layer (YSL) and it is not associated with heterochromatin. Although mH2A1 can exert a repressive function on specific differentially expressed genes at stages analysed, when found associated to these DNA sequences, it does not exert a general repressive function only based on its association with DNA. mH2A2 expression becomes evident at 24 hpf within the embryo body, without showing a clear effect on the transcription level of associated DNA. These results suggest that, contrary to the general notion, mH2A participation in lineage commitment is more active and complex than just repressing pluripotency genes. Our results also support the hypothesis that mH2A1 expression and chromatin binding is associated with the non-proliferating stage of the YSL nucleus without constitutively blocking the cell cycle. Our results do not support the hypothesis that mH2As have a specific role in repressing gene expression. Further investigation is needed to unveil specific mH2A DNA and protein interactions and their participation in the regulatory network controlling cell commitment, differentiation restriction, and/or cell cycle regulation at different developmental stages.

## Results

### Zebrafish mH2A1 and mH2A2 isoforms have different expression pattern during early embryo development

To investigate the expression of zebrafish mH2A isoforms during early embryogenesis we carried out quantitative RT-PCR in whole embryos to detect mH2A1 and mH2A2 during different developmental stages, and compare them to the expression of the pluripotency – associated transcription factors pou5f1/pou2 and nanog (zf orthologs of mammalian Pou5f1/Oct4 and Nanog) as markers of undifferentiated pre-segmentation shield/epiboly stages. As mentioned, mH2A1 is annotated to have two mRNA spliced variants^20^, h2afy-201 and h2afy-202, that are translated to the same mH2A1 protein which shows higher sequence homology to mammal mH2A1.1, thus we will designate here as mH2A1.1. We will label mH2A1 as the total mH2A1 coding mRNA expression when not distinguishing between putative transcript variants affecting coding exons.

**Figure 1:**
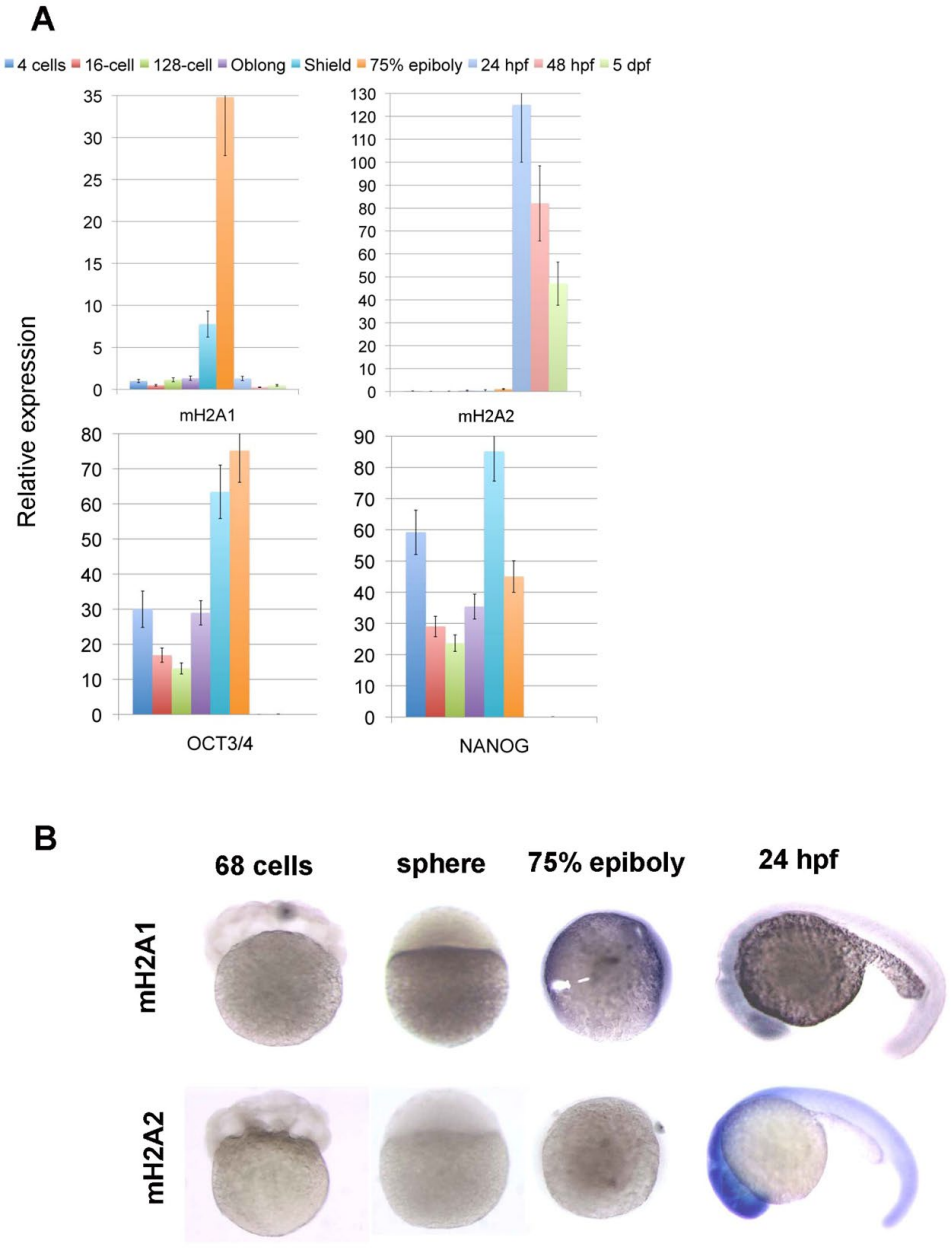
Zebrafish mH2A expression pattern during early embryo development. Wild type zebrafish embryos were collected at different developmental stages. (**A)** Quantitative RT-PCR was performed for mH2A1, mH2A2 and two genes characteristic of undifferentiated stage (OCT4 and NANOG). Mean values (n = 3) ± SEM are plotted. Values indicate relative expression of the specific gene normalized to GAPDH/TUBULIN. (**B)** Whole-mount RNA *in situ* hybridization using mH2A1 and mH2A2 specific probes showing different temporal and spatial gene expression pattern (n = 50–60 embryos/probe with identical labeling).

mH2A1 reaches maximum expression at shield (6 hpf) and 75% epiboly (8 hpf) stages decreasing during further embryo development, while mH2A2 increases progressively its expression during the same early embryo stages (Fig. 1A). We observed a clear shift in mH2A isoform prevalence after gastrulation, with mH2A2 becoming more prevalent than mH2A1 when segmentation and differentiation processes take place (Fig. 1A).

To analyse the expression pattern of mH2A isoforms we carried out wholemount *in situ* hybridization on zf embryos at different developmental stages. We confirmed that mH2A1 expression is highest at 75% epiboly stage, and intriguingly it localizes mainly in the cell layer surrounding the yolk sac, while mH2A2 expression start to be evident at 24 hpf and it is observed throughout the embryo body (Fig. 1B).

### mH2A1 and mH2A2 isoforms show different spatial and temporal expression, heterochromatin association, and DNA target genes during zebrafish embryogenesis

**Figure 2:**
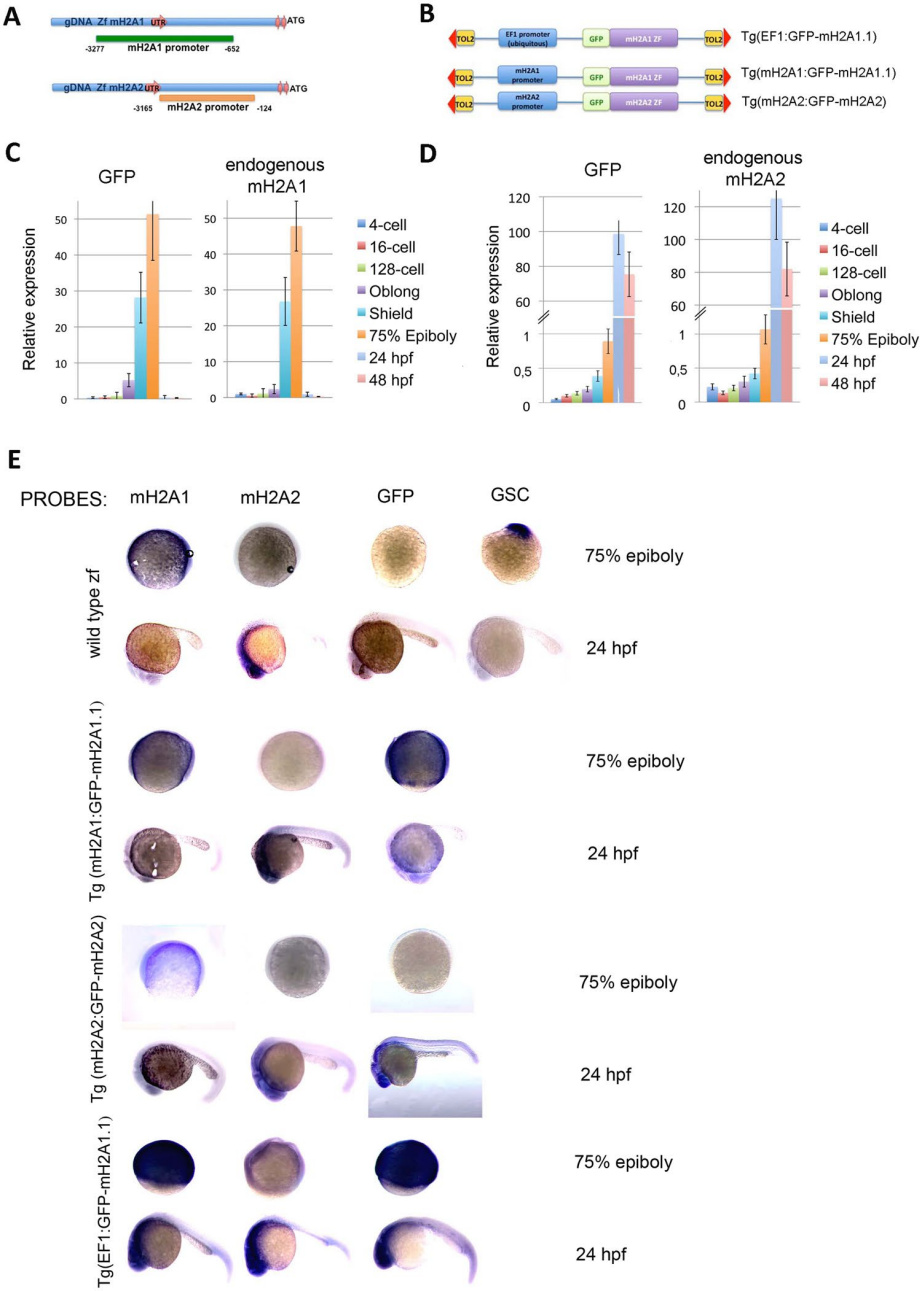
Identification and characterization of mH2A promoter regions and fusion proteins during embryogenesis. (**A)** Schematic representation of mH2A constructs structure/generated. The origin of mH2A UTR variants were determined from genomic DNA sequence. Identified promoter region responsible for transcription of each mH2A isoform are shown (promoter locations are indicated relative to ORF starting nucleotide triplet, ATG). (**B)** For mH2A reporter constructs mH2A1.1 and mH2A2 GFP-fusion proteins and either ubiquitous promoter (EF1) (EF1:GFP-mH2A1.1) or endogenous promoter (mH2A1:GFP-mH2A1.1 or mH2A2:GFP-mH2A2) were cloned into pTOL vector (Tol2 sequences that flank each transgene include the terminal inverted repeats (red arrowheads). (**C**,**D)** Identified mH2A promoter regions match endogenous expression. Expression analysis was conducted by injecting reporter constructs into the 1–2 cell stage of wt AB zebrafish embryos and collecting them at different development stages. Quantitative RT-PCR was performed to detect GFP and endogenous mH2A1 (**C**) or mH2A2 (**D**) expression. Mean values (n = 3) ± SEM are plotted. Values indicate relative expression of the specific gene normalized to GAPDH/TUBULIN. (**E**) Transgenic embryos for both Tg(EF1:GFP-mH2A1.1) and Tg(mH2A1:GFP-mH2A1.1) at two stages (75% epiboly and 24 hpf) were collected to perform whole-mount RNA *in situ* hybridization using mH2A1, mH2A2, GFP and GSC specific probes. (n = 50–60 embryos/probe with uniform labeling).

To further analyse the different expression level and localization of mH2A isoforms during early embryogenesis we first identified the promoter region that controls the expression of each isoform. We screened 5kbp upstream of the ORF starting codon (Fig. 2A) and cloned different PCR fragments into a modified Tol2 expression vector containing the EGFP reporter transgene. We tested 6 and 5 different fragments for mH2A1 and mH2A2 respectively using a GFP reporter expression assay that consisted on injecting each different construct into zf 1- or 2-cell stage embryos (Fig. S1). We then selected the construct with the promoter regions in which GFP expression most faithfully reflected the endogenous expression of each gene (75% epiboly for mH2A1 and 24 hpf for mH2A2).

We designed GFP:mH2A fusion proteins for each isoform to easily follow their protein expression *in vivo*, and cloned them both under the selected promoter region as well as or ubiquitous promoter (*Xenopus* EF1) (Fig. 2B) into the pTol2 vector.

To avoid variability due to transient construct embryo injection, we generated transgenic zebrafish expressing the fusion protein constructs controlled by either ubiquitous EF1 promoter (Tg(EF1: GFP-mH2A1.1) or the identified endogenous promoter Tg(mH2A1:GFP-mH2A1.1) and Tg(mH2A2:GFP-mH2A2).

To further validate our selected promoter regions for each mH2A isoform leading the expression of GFP fusion proteins, we first performed quantitative RT-PCR of transgenic zf embryos at different developmental stages. We found that GFP expression followed the same temporal expression pattern as the endogenous mH2A for both isoforms (Fig. 2C,D).

*In situ* hybridization using specific probes for mH2A1 and GFP also confirmed the precise RNA localization of the GFP-mH2A1 fusion construct i.e. the cell layer surrounding the yolk sac, while mH2A2 RNA detection became evident at 24 hpf within the embryo body. Another piece of evidence that indeed our promoter-driven GFP-mH2A1.1 was mimicking the endogenous expression of the gene and that no artifactual location was caused by fusion construct was the fact that when mH2A1.1 was expressed under the ubiquitous promoter, RNA was expressed throughout the whole embryo (Fig. 2E). mH2A2 and GSC probes were used to confirm probe specificity and developmental stage, respectively. Having identified mH2A isoform 1 and 2 promoter regions, and validated GFP fusion constructs that reproduce endogenous expression of these genes, we then tested if protein expression was also being faithfully reproduced.

**Figure 3:**
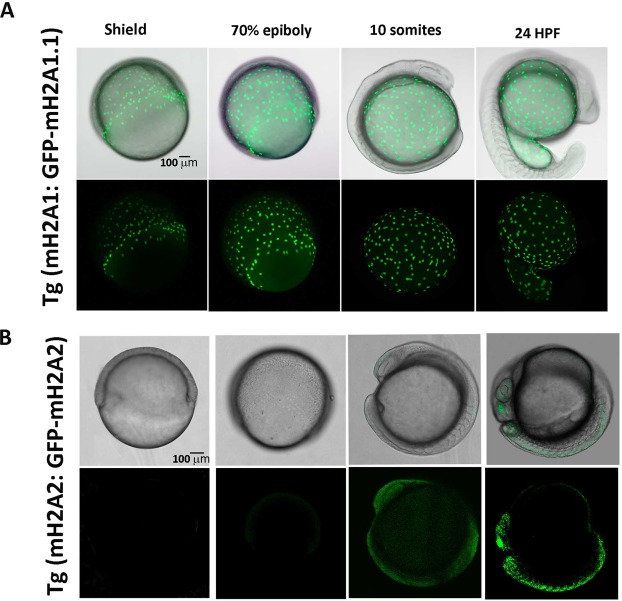
GFP-mH2A1.1 and GFP-mH2A2 protein expression under endogenous promoter during zebrafish embryogenesis show distinct temporal and location pattern. *In vivo* confocal imaging of transgenic zf embryos expressing mH2A1:GFP-mH2A1.1 (**A**) or mH2A2:GFP-mH2A2 (**B**) reveals that mH2A1 is expressed in yolk syncytial layer (YSL) while mH2A2 is expressed within the embryo body becoming evident at 10 somites and 24 hpf stages. Figure shows a representative series of GFP and merged brightfield-GFP snapshots at different developmental stages during live image capturing. (Assay conducted in triplicate with n = 12 embryos/isoform).

**Figure 4:**
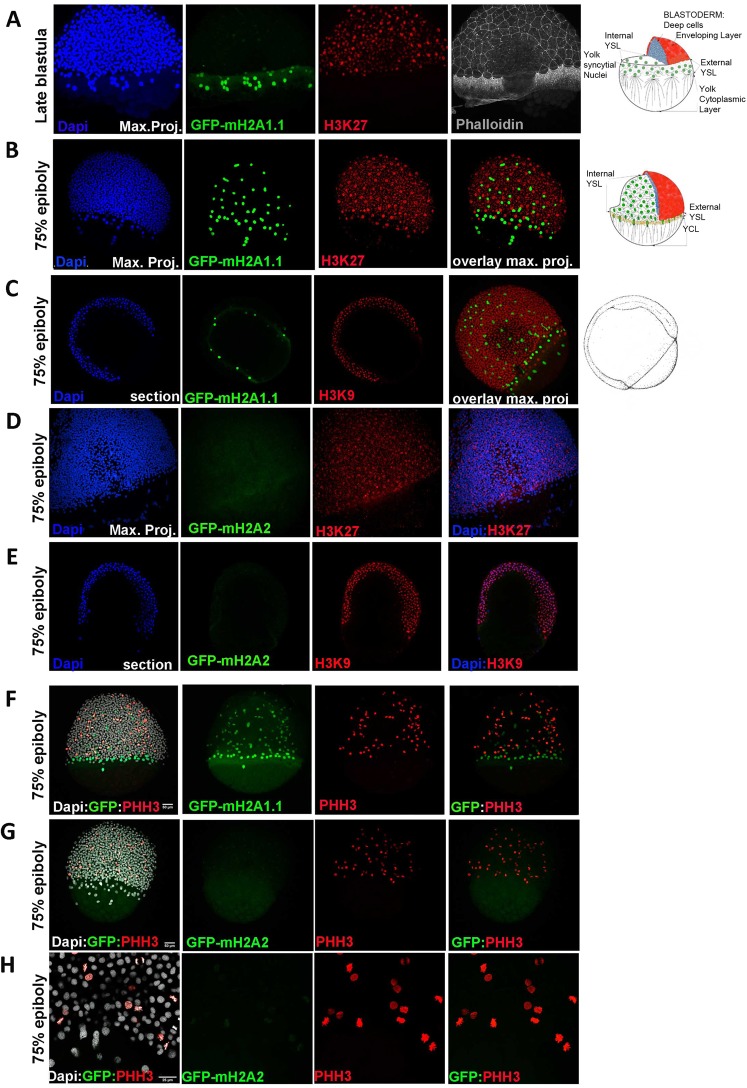
mH2A isoforms differential colocalization with heterochromatin and mitotic marks in zebrafish embryos at epiboly stage. Transgenic zf embryos expressing mH2A1:GFP-mH2A1.1 (**A–C**,**F**) or mH2A2:GFP-mH2A2 (**D**,**E**,**G**,**H**) at late blastula (**A**) or 75% epiboly (**B–H**) stages were analysed using immunohistochemistry to detect the heterochromatin markers trimethyl Histone3 lysine K27 and K9 (H3K27me3 and H3K9me3) (**A–E**) or mitotic nuclei phosphohistone H3 (pHH3) (**F–H**). DAPI and Phalloidin-conjugate were used for nucleus and cytoskeleton labelling respectively. (**A–C)** Confocal microscope image projection of late blastula (**A**) and 75% epiboly stage **(B)** transgenic **mH2A1:GFP-mH2A1**.**1** embryo shows YSL localization of GFP positive cells and lack of colocalization with H3K27me3. Apical pole and vegetal pole are pointed in the image. To the right of (**A–C)** there is a schematic illustration of the organization of the cortical cytoplasm of the yolk cell in relation to other cell types in zebrafish embryo during epiboly (draws at late blastula and 60% epiboly respectively). (**C**) Confocal microscope image section also shows absence H3K9m3 signal in the nuclei of YSL cells, where GFP-mH2A1.1 is expressed. (**D**,**E)** Confocal microscope image projection (**D**) and section (**E**) of 75% epiboly stage transgenic **mH2A2:GFP-mH2A2** embryo stained with heterochromatin markers H3K27me3 and H3K9me3 shows heterochromatin labelling in the whole embryo while GFP (mH2A2) expression is still very weak. (**F–H)**. Confocal microscope image projection of 75% epiboly stage transgenic **mH2A1:GFP-mH2A1**.**1 (F) or mH2A2:GFP-mH2A2 (G**,**H)** with labelled pHH3 mitotic nuclei. Figure show non-mitotic mH2A1 positive YSL nucleus and proliferative cells within the embryo body. (**H)** Higher magnification of images of **mH2A2:GFP-mH2A2 transgenic fish** clearly showing pHH3 positive cells while there is a low level GFP-mH2A2 expression (**H**). Each labelling assay was conducted in triplicate with n = 40–50 embryos/immunolabeling.

To confirm our *in-situ* RNA expression data, we analysed our transgenic lines at the protein level, checking the expression time and localization of the fusion proteins by fluorescence imaging of zf embryos at selected developmental stages for both isoforms (Figs 3A,B and S2). *In vivo* confocal imaging of transgenic zf embryos expressing GFP-mH2A1.1 revealed that mH2A1.1 protein is expressed in the cell layer that surrounds the yolk sac - called yolk syncytial layer (YSL) (Fig. 3A and Supplementary Video 1) - throughout embryo development, while GFP-mH2A2 is expressed in embryo proper, becoming evident at 24 hpf, and not in the yolk sac (Fig. 3B and Supplementary Videos 2 and 3). Maximal intensity projection of confocal images of fixed embryos at late blastula and 75% epiboly stage, showed GFP-mH2A1.1 fusion protein expression at both internal and external YS layers (Fig. 4A,B).

**Figure 5:**
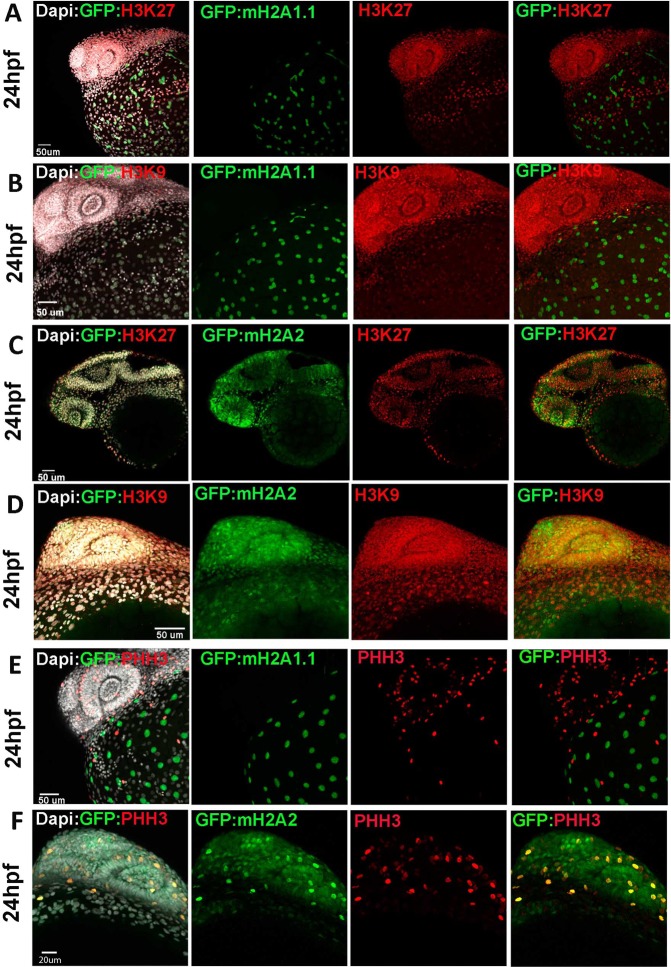
mH2A isoforms differential colocalization with heterochromatin and mitotic marks in zebrafish embryos at 24 hpf stage. Transgenic zf embryos expressing mH2A1:GFP-mH2A1.1 (**A**,**B**,**E**) or mH2A2:GFP-mH2A2 (**C**,**D**,**F**) at 24 hpf stage were analysed using immunohistochemistry to detect heterochromatin markers trimethyl Histone3 lysine K27 and K9 (H3K27me3 and H3K9me3) (**A–D**) or mitotic nuclei Phosphohistone H3 (pHH3) (**E**,**F**). DAPI was used for nucleus labelling. Confocal microscope image projection of 24 hpf transgenic fish. (**A**,**B**) **mH2A1:GFP-mH2A1**.**1** embryo shows YSL localization of GFP positive cells and lack of colocalization with H3K27me3 (**A**,**B**) and H3K9m3 (**C**) throughout the embryo body. (**C**,**D) mH2A2:GFP-mH2A2** embryo stained with heterochromatin markers H3K27me3 (**D**) and H3K9me3 (**E**) reveals areas of colocalization. (**E**,**F) mH2A1:GFP-mH2A1**.**1 (E) or mH2A2:GFP-mH2A2 (F)** with labelled pHH3 mitotic nuclei shows partial colocalization of GFP-mH2A2 isoform with mitotic nuclei. Each labelling assay was conducted in triplicate with n = 40–50 embryos/immunolabeling.

In order to check if mH2A isoforms localize preferentially in condensed chromatin, we performed immunocytochemistry to detect either H3K27me3 or H3K9me3 heterochromatin markers at 75% epiboly (Fig. 4) or 24 hpf (Fig. 5) stages. We observed that YSL nucleus expressing GFP-mH2A1.1 do not colocalize with heterochromatin markers that stain most nuclei of the developing embryo (Figs 4A–C, 5A,B) while GFP-mH2A2 colocalization with both H3K27me3 and H3K9me3 in the embryo body becomes more evident albeit not complete, at 24 hpf. At earlier stages, GFP-mH2A2 expression is hardly detectable (Figs 4D,E and 5C,D).

We confirmed that GFP-mH2A1.1 fusion protein does not have artifactual binding properties and can bind normally to heterochromatin. Ubiquitously expressed (EF1 promoter) GFP-mH2A1 protein colocalize with the heterochromatin marker H3K27me3 in transgenic zf Tg(EF1:GFP-mH2A1.1) (Fig. S3A).

Since mH2A has been associated with differentiating cells and its downregulation is associated with cancer initiation and progression^23,24^, we checked if mH2A isoforms are associated with quiescent or proliferating cells in these early stages of development (75% epiboly and 24 hpf) we performed immunohistochemistry to detect phosphohistone H3 (pHH3) as marker for nuclei undergoing mitosis. Previous results show that YSL nuclear divisions cease around the sphere stage of development (4 hpf)^25,26^. Our results confirm the low proliferating rate of YSL at 75% epiboly and 24 hpf stages, and that GFP-mH2A1.1 shows no colocalization with mitotic pHH3 positive nucleus (Figs 4F and 5E). In contrast, mH2A2 is expressed in both dividing and not dividing cells in the embryo at 24 hpf (Fig. 5F) Although mH2A1 evidence can suggest that absence of expression of endogenous mH2A1 correlate with cell division, images of transgenic embryos expressing mH2A1-GFP ubiquitously, Tg(EF1:GFP-mH2A1.1), show that mH2A1.1 expression does not directly impede cell mitosis within the embryo body at early stages of development (Fig. S4) where it colocalizes with mitotic pHH3 positive cells.

### mH2A1 and mH2A2 are associated with different genes throughout development

**Figure 6:**
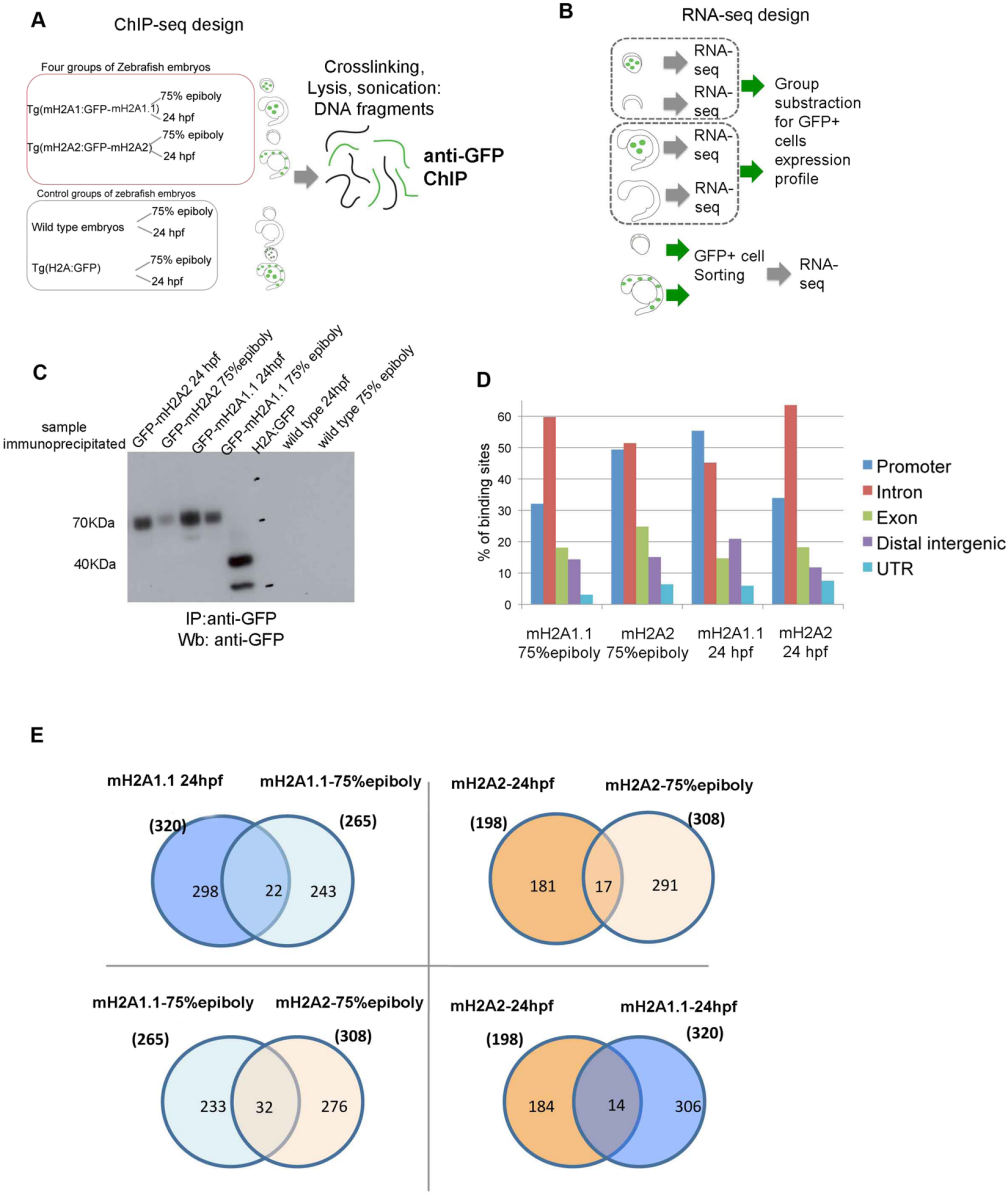
mH2A1.1 and mH2A2 proteins are associated with different genes during early zebrafish embryogenesis. (**A)** Scheme of samples and control used for ChIP with anti-GFP antibody. (**B**). Scheme of RNA-seq experimental design. (**C)** Immunoprecipitation (IP) and western blot (Wb) using specific antibodies against GFP show specific 70 KDa band for fusion proteins in lysates from transgenic fishes Tg(mH2A1:GFP-mH2A1.1) and Tg(mH2A2:GFP-mH2A2) at selected development stages, and 40KDa band for GFP-expressing transgenic fish Tg(H2A:GFP). Wild type-fish lysates were used as negative controls. (**D)** Analysis of the identity of genome regions bound with different mH2A isoforms at 75% epiboly and 24 hpf stages using ChIP-seq data. (**E)** Venn diagrams of ChIP-assay list of genes showing the number of specific and overlapping target genes identified for GFP-mH2A1.1 and GFP-mH2A2 fusion proteins at different embryo stages (75% epiboly and 24 hpf).

To identify the genomic regions that mH2A1.1 and mH2A2 - expressed under endogenous promoter - were associated with, we performed chromatin immunoprecipitation using a ChIP-grade anti-GFP antibody and genomic analysis (ChIP-seq) at two developmental stages, pre-segmentation (gastrula: 75% epiboly) and segmentation stage (24 hpf) (Fig. 6A,C). In Supplementary Fig. S6, we show sample peaks and areas around them using the Integrative Genomics Viewer (IGV) for both isoforms at the two time points^27^. The sample peaks show clear overrepresentation of the binding regions by the reads compared to their flanking regions.

In Tables S1–S4, we list the genes targeted by the two isoforms at 75% epiboly and 24 hpf following the ChIP-seq analysis. We categorized the binding regions of the isoforms based on genome annotation (promoter, intron, exon, distal and UTR regions) and observed that the promoter and intron regions were the most preferred binding regions for both of the isoforms at both of the stages analysed (Fig. 6D). Based on *regioneR* statistical association analysis, in all four cases, the peaks were significantly associated with the promoter, intron and exon regions (p < 0.05) but not with the distal intergenic and the UTR regions.

When we compared the genes targeted by the two isoforms at the two time points, our Venn diagram analysis showed that, interestingly, not only mH2A1.1 and mH2A2 are associated with different target DNA regions, but also that each isoform’s binding targets change dramatically during gastrulation until the segmentation stage, thus suggesting a highly specific regulation of mH2A localization during zf embryogenesis (Fig. 6E).

Functional analysis and classification of target genes revealed their association with developmental processes (Table 1). Interestingly, only mH2A1.1 shows previously described^10,16,19^ targeting of HOX family members at 24 hpf stage (Table S7) and poor representation of Wnt signalling, FGF signalling or GATA genes for both of the isoforms at both of the stages. We did not identify occupancy of pluripotent regulatory network genes (Oct4, Sox2 or Nanog) at the selected stages^16^. Canonical pathway and molecular network analysis identified possible specific pathways regulated by mH2A isoforms at different stages (Tables S5, S6). Among others, cAMP- mediated, Rho, PKA, Netrin and nNOS signalling pathways appear as regulated by mH2A1 at 75% epiboly. Complement system and transcriptional regulatory network of ESC pathways as well as cAMP-mediated and Rho signalling pathways are represented at 24 hpf. Calcium, Netrin, GP6, ErbB4, neuregulin, GABA receptor or nNOS signalling appear as associated pathways of mH2A2 bound genes at 75% epiboly while pyrimidine processing, sumoylation or cell cycle checkpoint control and mismatch repair pathways are mostly represented at 24 hpf.

**Table 1:** Summary of functional analysis of ChIP-seq analysis.

IPA® analysis of ChIP data					
Physiological System Development and Function			Molecular and Cellular Functions		
Name	p-value	#Molecules	Name	p-value	#Molecules
mH2A1.1-75% epiboly			mH2A1.1–75% epiboly		
Nervous System Development and Function	7,34E-03–1,83E-06	45	Cell Morphology	7,87E-03–1,09E-06	44
Organismal Development	6,36E-03–1,83E-06	45	Cellular Development	8,43E-03–1,09E-06	49
Tissue Development	8,43E-03–1,83E-06	41	Cellular Assembly and Organization	8,13E-03–1,83E-06	38
Embryonic Development	6,10E-03–7,98E-06	38	Cellular Function and Maintenance	7,08E-03–1,83E-06	38
Connective Tissue Development and Function	8,43E-03–3,69E-05	17	Cellular Growth and Proliferation	7,34E-03–1,83E-06	43
mH2A1.1–24 hpf			mH2A1.1–24 hpf		
Embryonic Development	7,08E-03–5,03E-15	57	Gene Expression	1,39E-03–2,16E-06	29
Organismal Development	7,75E-03–5,03E-15	77	Cellular Development	6,43E-03–4,42E-05	28
Connective Tissue Development and Function	7,75E-03–3,80E-13	26	Cellular Growth and Proliferation	6,05E-03–9,34E-05	23
Organ Development	7,00E-03–3,80E-13	50	Cellular Movement	6,05E-03–1,68E-04	37
Skeletal and Muscular System Development and Function	7,75E-03–3,80E-13	37	Cell-To-Cell Signaling and Interaction	6,05E-03–3,44E-04	13
mH2A2–75% epiboly			mH2A2–75% epiboly		
Organismal Survival	5,15E-04–1,94E-06	47	RNA Post-Transcriptional Modification	1,07E-03–4,47E-05	10
Embryonic Development	7,25E-03–1,41E-05	41	Gene Expression	6,05E-03–1,44E-04	41
Organismal Development	7,25E-03–1,41E-05	65	Cell Morphology	7,25E-03–1,50E-04	37
Tissue Development	7,25E-03–1,41E-05	50	Cell-To-Cell Signaling and Interaction	7,25E-03–1,56E-04	19
Tissue Morphology	7,25E-03–1,41E-05	30	Protein Synthesis	4,46E-03–2,75E-04	23
mH2A2–24 hpf			mH2A2–24 hpf		
Organismal Functions	1,55E-02–4,77E-05	11	Amino Acid Metabolism	2,93E-02–7,24E-05	2
Nervous System Development and Function	2,45E-02–1,34E-03	16	Drug Metabolism	2,45E-02–7,24E-05	4
Organ Morphology	2,93E-02–1,34E-03	20	Molecular Transport	2,93E-02–7,24E-05	6
Organismal Development	2,93E-02–1,34E-03	34	Small Molecule Biochemistry	2,93E-02–7,24E-05	12
Embryonic Development	2,93E-02–4,10E-03	27	Vitamin and Mineral Metabolism	2,93E-02–7,24E-05	4

Functional analysis of target genomic regions bound to mH2A1 and mH2A2 isoforms at 75% epiboly and 24 hpf stages using IPA® software analysis.

To determine if the targets of mH2A isoforms are related to the transcriptional activity of the genes the isoforms are found to be associated with, we performed RNA-seq analysis of cells expressing either GFP-mH2A1.1 or GFP-mH2A2 at both of the developmental stages. For GFP-mH2A2 samples, GFP-positive cells were isolated and RNA was used for sequencing. Since GFP-mH2A1.1 is expressed in YSL, we could not perform cell sorting, and average expression data of GFP positive YSL cells was obtained by subtracting the RNA expression profile of deyolked embryos (embryos for which their yolk sac, and thus GFP positive cells, were removed) from the RNA expression profile of whole embryos (Table S8) (Fig. 6B).

In order to correlate the ChIP-seq and RNA-seq analysis results, we grouped target genes specific to each isoform and stage into eight categories based on their relative expression levels (Fig. S7). The results indicate that mH2A1 exerts a downregulatory effect on its target genes at 75% epiboly stage when compared both with 24 hpf (Fig. S7A) and with mH2A2 (Fig. S7C). The mH2A2 isoform does not have as clear pattern as mH2A1, showing mild average upregulation of target genes during development and when compared with mH2A1 (Fig. S7B–D).

To further study the effect of mH2A binding on gene expression, we analysed the effect of isoform binding on expression level, using RNA-seq TPM (transcripts per million) data. For each gene, we calculated the z-score of its log-transformed TPM value, i.e., we identified the number of standard deviations the gene’s expression value is away from the average expression value seen in a given sample (Fig. S8, Tables S1–S4). We then considered four intervals for z-values to assess if the genes have high or low expression values. When we calculated the percentage of genes in each interval for the target genes and compared this with the corresponding percentage values for all genes measured by the RNA-seq platform, we observed that mH2A1.1 targets tend to be enriched in the low expression intervals and depleted in the high expression intervals. An opposite trend holds true for mH2A2 targets with the exception that the enrichment in high expression intervals are observed only for the 75% epiboly stage but not the 24 hpf stage. Although these results suggest that mH2A1.1 and mH2A2 may have a general down- and up-regulation effect after binding to DNA, respectively, we note that this is just a general trend in expression of the targets of the two isoforms. There are are highly expressed target genes and lowly expressed target genes for each of the two isoforms. Therefore, the repressing or activating effect of the isoforms depends on the specific molecular environment associated with cell location, stage and interaction network during development.

RNA-seq results were used to functionally annotate the genes differentially expressed by GFP-mH2A1.1 or GFP-mH2A2 positive cells at both developmental stages, using Ingenuity® Pathway Analysis (IPA®) Software (Table 2). Table 2 shows top represented canonical pathway groups (extended at Supplementary Table S9) and molecular and cellular function (extended at Supplementary Tables S10, S11) within each isoform comparison.

**Table 2:** Summary of functional analysis of RNA-seq relative expression data.

IPA® analysis summary of RNA-seq data					
Top Canonical Pathways			Molecular and Cellular Functions		
mH2A1.1–24 hpf vs mH2A1.1–75%epiboly			mH2A1.1–24 hpf vs mH2A1.1–75%epiboly		
Name	p-value	overlap	Name	p-value	N° of molecules
Sirtuin Signaling Pathway	1,57E-19	29,5% 86/292	Cell Death and Survival	4,71E-07–4,65E-51	909
Oxidative Phosphorylation	1,38E-18	43,1% 47/109	Gene Expression	6,73E-08–2,10E-35	622
Mitochondrial Dysfunction	2,35E-17	34,5% 59/171	RNA Post-Transcriptional Modification	3,10E-09–3,51E-25	114
EIF2 Signaling	2,51E-12	26,9% 61/227	Cell Cycle	3,19E-07–4,66E-25	427
RhoGDI Signaling	3,76E-10	27,1% 48/177	Cellular Development	3,27E-07–1,66E-24	788
mH2A1.1–75%epi vs mH2A2–75% epiboly			mH2A1.1–75%epiboly vs mH2A2–75% epiboly		
FXR/RXR Activation	1,96E-05	4,8% 6/126	Molecular Transport	2,78E-03–2,95E-10	45
Unfolded protein response	1,07E-04	7,1% 4/56	Lipid Metabolism	2,48E-03–3,99E-08	29
Glucocorticoid Receptor Signaling	1,40E-04	2,3% 8/345	Small Molecule Biochemistry	2,48E-03–3,99E-08	41
Glycolysis I	1,98E-04	11,5% 3/26	Energy Production	1,38E-03–6,92E-07	11
Gluconeogenesis I	1,98E-04	11,5% 3/26	Carbohydrate Metabolism	2,78E-03–9,03E-06	20
mH2A2–24 hpf vs mH2A1–24 hpf			mH2A2–24 hpf vs mH2A1–24 hpf		
Axonal Guidance Signaling	2,85E-13	18,2% 83/457	Cell Death and Survival	1,88E-12–5,85E-38	676
Mitochondrial Dysfunction	2,23E-10	23,4% 40/171	Cellular Movement	7,86E-09–5,35E-35	488
Sirtuin Signaling Pathway	2,60E-10	19,2% 56/292	Cellular Assembly and Organization	2,57E-09–7,77E-28	408
Oxidative Phosphorylation	6,27E-10	27,5% 30/109	Cellular Function and Maintenance	1,41E-09–7,77E-28	522
Signaling by Rho Family GTPases	6,95E-10	19,8% 50/252	Gene Expression	1,14E-20–1,73E-25	422
mH2A2–24 hpf vs mH2A2–75% epiboly			mH2A2–24 hpf vs mH2A2–75% epiboly		
EIF2 Signaling	3,72E-31	49,3% 112/227	Gene Expression	6,89E-12–7,50E-58	971
Axonal Guidance Signaling	6,90E-25	35,9% 164/457	Cell Death and Survival	2,41E-12–8,16E-57	1315
Regulation of eIF4 and p70S6K Signaling	1,55E-16	43,6% 71/163	Cellular Assembly and Organization	1,49E-11–5,68E-35	749
Sirtuin Signaling Pathway	4,44E-15	34,9% 102/292	Cellular Function and Maintenance	1,91E-13–5,68E-35	952
Molecular Mechanisms of Cancer	3,93E-14	31,5% 124/394	Cellular Movement	4,74E-13–1,14E-33	88

Functional analysis of differentially expressed genes between cells expressing mH2A1 and mH2A2 at different developmental stages (75% epiboly and 24 hpf) using Ingenuity software analysis. Only the top five represented categories are shown.

Cells expressing mH2A1.1 during transition from epiboly to segmentation show a change in transcription profile associated with cell cycle regulation (decrease of expression of CDK9, 11, 12, 13, CCNE1, CCNE2, CCND1 among others) and Sirtuins (increase of SIRT2, SIRT5), and other canonical pathways such as mitochondrial function (increase in mitochondrial network and function components as slc25a4, mcu, aifm3, gpam mss51, oxsm, atp5j2, micu1, tomm5 among others) or increase in EIF2 signalling pathway components (as RAP1B; RPS6,7,9,23; RPL 9,19,30,35…) and RhoGDI signalling pathway (Rnd2, ARHGEF10,19; ARHGDIA; PRKCA, SRC). Also, HOX, WNT, SOX, BMP and GATA family of genes suffered marked regulation during gastrulation to segmentation transition in mH2A1.1 expressing cells (Table S8) supporting the important role of YSL in signalling for early embryonic development.

Cells expressing mH2A2 during this developmental period, undergo transcriptional changes more related to cellular movement, assembly and organization and cell death through factors involved in EIF2, Axonal guidance, regulation of eIF4 and Sirtuin signalling among others, including also the regulation of HOX, WNT, SOX, BMP, SMAD and GATA family of genes (Tables 2 and S9–S11). Indicating that although, as expected, these major developmental signalling pathways are regulated during gastrulation to segmentation stages, this is not due to direct interaction of mH2A isoforms with their regulatory regions.

At the same developmental stage, when we compared cells expressing mH2A1.1 or mH2A2, we saw differences in transcriptional profile related to different metabolism and molecular transport activated pathways. At 75% epiboly stage, there is a clear upregulation of the group of membrane transport proteins (SLCs), FABP1 and FABP2, and Annexins in mH2A1.1 expressing cells, while at 24 hpf they differ more in pathways related to cellular movement, assembly and organization, and cell death through factors involved in axonal guidance, Sirtuins, Rho GTPases signalling, and mitochondrial oxidative phosphorylation pathways. Cells expressing mH2A2 at 24 hpf showed upregulation of members of the HOX gene family (e.g. Hoxa2a, a4b, a9a, a10b, a11b, a13a, Hoxb2a, b3a, b4a, b6a, Hoxc1a, c4a, c10a, c11b, c12a, c13a, d3a, d4a, d9a, d11a, d12a), also of the Wnt gene family (e.g. wnt7aa, wnt7bb, wnt4a, wnt11r or wnt3), and of the SOX gene family (e.g. Sox1, Sox2, Sox3 Sox4a, Sox6, Sox7, Sox9b, Sox10). Only Sox17 and Sox32 were downregulated in mH2A2 cells when compared to mH2A1.1 expressing cells at 24 hpf. These results provide strong evidence of activation of differentiation pathways at this stage of development within the embryo body of zebrafish. Regarding the GATA family genes, only Gata1 was upregulated in mH2A2 expressing cells while Gata2a, 3, 5, and 6 were upregulated in mH2A1 positive cells underlining the role of YSL in hematopoietic and endodermal differentiation signalling. Of note, crucial Nodal signalling pathway molecules Lefty1, 2 and FoxH1 were upregulated in mH2A1.1 expressing YSL, again supporting the influence of this extraembryonic layer on embryonic layers patterning.

### Both mH2A1 and mH2A2 are required for proper zebrafish embryo development

**Figure 7:**
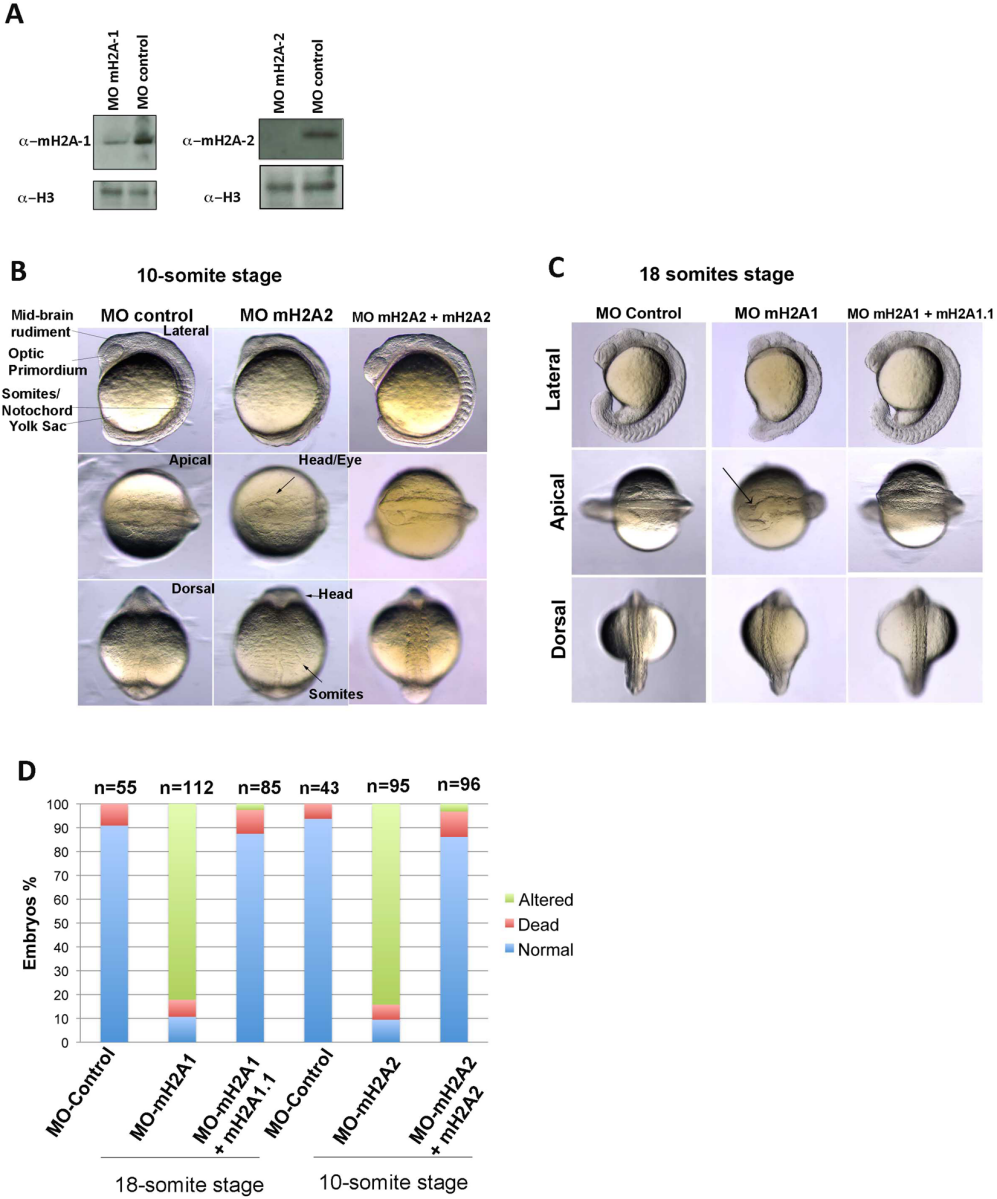
mH2A2 and mH2A1 are required for proper zebrafish embryo development. (**A)** An inhibitory morpholino (MO) was designed against the translational start site of zebrafish mH2A1 or mH2A2 mRNA (sequences at Supplementary Table S12). For the control mismatch morpholino we introduced a few base alterations. Total nuclei of zebrafish embryos were analysed by anti-mH2A1 or anti-mH2A2, and anti-histone H3 immunoblotting after specific morpholino injection. Full-length blots are presented in Supplementary Fig. S11. (**B)** Characterization of mH2A1 morphants and control embryos at the 18-somite stage. Lateral, apical and dorsal view of fixed zebrafish embryos show alterations in midbrain structure and organization (arrow). Somite number was correct, but their distribution along the embryo was shortened. Yolk sac extension and caudal fin was not properly formed after mH2A1 downregulation. In all experiments we co-injected with a morpholino against p53 (p53 MO) to mitigate the nonspecific dose-dependent neural toxicity widely reported^56^. Rescues were generated by injection of embryos with mH2A1 morpholino in combination with mH2A1.1 mRNA. (**C)** Characterization of mH2A2 morphants and control embryos at the 10-somite stage. Lateral, apical and dorsal view of fixed zebrafish embryos show alterations in midbrain, optic primordium and somites and fin formation/structure after mH2A2 downregulation. In all experiments we co-injected with a morpholino against p53 (p53 MO) to mitigate the nonspecific dose-dependent neural toxicity widely reported^56^. Rescues were generated by injection of embryos with mH2A2 morpholino in combination with mH2A2 mRNA. (**D)** Stacked bar graph representing the percentage of embryos which are normal, dead or showing described altered phenotype after injecting control and mH2A1 and mH2A2 morpholinos, and rescued with mH2A1.1 or mH2A2 mRNA at the developmental stages shown in figure (**C**), where n represents numbers of embryos injected.

To test the role of mH2A isoforms *in vivo*, we downregulated protein expression of both isoforms using specific morpholino antisense oligos (MOs) targeting the translational start site of the mRNA (Fig. 7A). Supporting previously published data^19^, that showed that injection of mH2A2 MO resulted in developmental abnormalities and PAX2A incomplete labelling at 11-somites stage, our data show that at the 10-somites stage (14 hpf), embryos showed mid-brain size reduction, somites disorganization and fin underdevelopment (Fig. 7B). Our data also show that at 24 hpf, the 4^th^/ hindbrain ventricle cavity was drastically reduced in size and optic tectum also showed diminished size. The rostral telencephalon appears unstructured after mH2A2 knockdown as well (Fig. S5A).

We confirmed already published PAX2A incomplete labelling after mH2A2 downregulation, performing *in situ* staining of this regulator in the genetic cascade controlling mid-hindbrain development, at 18-somites stage (16 hpf) indicating a defect in the formation of the midbrain-hindbrain boundary (MHB), and thus formation, of this structure after not only mH2A2 but also mH2A1 downregulation (Fig. S5B).

Downregulation of mH2A1 also affected proper brain development, with rostral telencephalon unstructured organization and mesencephalon and cerebellum formation delay at 18-somites stage (Fig. 7C), again confirming defects in midbrain formation, however, no effects on somites organization were seen. Downregulation of mH2A1 also affected yolk sac extension, decreased somite size, and almost no formation of a distal fin (Fig. 7C).

## Discussion

In this study, we have explored mH2A isoforms’ expression and function during early embryogenesis using zebrafish as a model organism. We have provided compelling evidence showing distinct temporal and spatial expression patterns, genome association regions, and associated global RNA expression profiles during zebrafish embryogenesis, suggesting that they have different developmental roles.

There is limited information addressing the expression of mH2A during early embryonic development^8,9,13^. Chang *et al*. first studied mH2A1 behaviour during mouse oocyte maturation and preimplantation embryos, showing that maternal mH2A1.2 store locate at both centrosomes and heterochromatin, it is removed soon after fertilization and protein expression reappears in embryos after the 8-cell stage and persists in morulae and preimplantation blastocysts^13^.

Pasque and colleagues showed that in mouse early blastocyst (E3.5) prior to implantation, nuclear mH2A1 was detected at equivalent levels in cells of the trophectoderm and those of ICM, while in E4.5 (late blastocyst - postimplantation) mH2A1 was only present in trophectoderm layer, and highly expressed thereafter in extra embryonic ectoderm and endoderm and not in the epiblast. Total mH2A1 (mH2A1.1 and mH2A1.2, as they did not distinguished between spliced isoforms in this study), progressively appeared in all somatic cells of E9.5 embryos. This observation is partially in agreement with our results, showing that zebrafish mH2A1, highly homolog to mouse and human mH2A1.1, is expressed mainly in the extraembryonic YS layer during zf development, however, we found that mH2A1.1 plays a minimal role in zf late embryo and adult individuals, possibly been replaced mH2A2^8,9^.

Expression and localization studies in zebrafish during oocyte maturation and early development would help understand similarities or differences between species and the role of mH2A1.1 isoform in this process.

To the best of our knowledge, there is only one report describing the role of mH2A isoforms in zebrafish^19^. This work shows that, in contrast to many human cell types, zebrafish embryos expressed predominantly mH2A2, whereas mH2A1 was hardly detectable. The data reported by Buschbeck and colleagues was acquired 24 hours post fertilization, but no information is provided for earlier stages. Our work shows differential dynamics and roles of mH2A isoforms during these stages, and unveils the need for further functional analysis of mH2A isoforms during zebrafish early development.

Extraembryonic structures regulate early embryonic patterning in many vertebrates^28–30^. The extraembryonic YSL in zebrafish is believed to be equivalent to mouse visceral endoderm^28^ and it is reported to have important roles in germ layer patterning in zebrafish. It is also required for the formation of ventrolateral mesendoderm, and induction of the nodal-related genes in the ventrolateral marginal blastomeres^31^. Our results strongly suggest that in the YSL, mH2A1 mediated signaling may play an important role in the formation of brain structures.

Based on mouse studies, mH2A1 has been associated with markers of heterochromatin such as H3K9me3 and H3k27me3 in the inactive X chromosome of trophoblast cells^8^. Others have shown that mH2A proteins are required for maintaining proper heterochromatin architecture through the binding of Lamin B1 to H3K9me3-marked repeats^32^ in transformed HepG2 human cell lines. Also, ChIP-seq data from mouse and human pluripotent cells^11,16^ indicate that mH2A isoforms occupy genes that are marked with H3K27me3 and then expressed at low levels, suggesting that mH2A repressive function could be associated with H3 modifications. However, in the zebrafish extraembryonic YSL, where mH2A1 is expressed, we observed low levels of H3K27me3 and H3K9me3 suggesting that, if mH2A1 plays a repressive role, other mechanisms must be at play.

This finding also implies that zf YSL and mouse trophectoderms are not functionally equivalent, and that mH2A1 association with DNA is not necessarily forming heterochromatin, rising more questions about its regulatory function.

Our results do support the hypothesis that mH2A1 expression is associated with non-proliferating cells in YSL however, our data from transgenic fish ubiquitously expressing mH2A1 indicates that mH2A1 is not necessarily causing this proliferation blockage as when mH2A1 is expressed within the embryo body, cells can proliferate normally. We cannot discard the possibility however, that mH2A1 participates in mitosis interruption when specific molecular environment and/or interactions govern a specific cell stage or location.

This hypothesis is in accordance with mH2A role inhibiting certain types of cancer progression^33–35^ that is supposed to be due to, at least in part, cell cycle *G1/S transition* inhibition via regulation of CDKs (CDK 4, 6 and 8) and cyclins (Cyclin D1 and D2)^36^. Our analysis suggests that mH2A1 may be differentially controlling cell cycle progression at different embryo stages. During gastrulation, mH2A1 is associated with cell cycle regulators such as Pka, SLIT1, Sos, MED13 or ERK1/2, which are probably associated with the proliferation blockage these YSL cells suffer, and with the decreased expression of specific CDKs and CCNs observed at 24 hpf.

Our results support the theory that mH2A’s role in inactivation and gene silencing may only apply to certain cell types and developmental stages of the embryo, such as X-chromosome inactivation in mammals^4,5,8^. We found that in some cells, mH2A is not associated with the heterochromatin, and that it can regulate gene transcription by both activating and repressing it^37–40^. The specific effect may be dependent on the physical and functional interactions with specific regulators (e.g. HDAC1/2, PARP1 or Pbx1 as previously reported) that are recruited by mH2A1 to control gene expression^1,19,38,39,41^.

We have identified sets of target DNA regions for mH2A1 and mH2A2 isoforms that confirm their relationship with developmental processes, but show different subsets of target genes to the ones previously described^10,19^. Organ, nervous system, and skeletal and muscular development appear as important networks represented with specific categories and target genes depending on isoform and developmental stage (Table S7) while Wnt, FGF or PAX signaling^10,19^ do not have major representation. TGFb and cell-to-cell or matrix anchorage and signalling molecules are present among the associated DNA sequences.

More tissue specific knock down studies are needed to unravel the different roles mH2A isoforms have during embryo development. Nonetheless, our morpholino-mediated knockdown experiments indicate that both isoforms play a critical role in early development and differentiation, especially in brain areas. These results support previous studies done in zebrafish^10,19^, where brain development defects arised after mH2A2 MO downregulation, and mH2A knockdown mouse embryonic stem cells (mESC)^10,16^. However, Pehrson *et al*.^42^ did not see evidence that mH2As have a crucial role in differentiation or morphogenesis during embryonic development, although they saw a decrease in pups’ size and weight, and increased perinatal death that could involve developmental defects that are not morphologically obvious. Beside this apparent incoherence, there is also the possibility that mH2A has an essential developmental role in some species, with less redundant proteins, zebrafish would be then an adequate model to study the role of these proteins during development.

It seems likely that the findings with macroH2A KO experiments are particularly relevant to closely related vertebrate species. The apparent loss of mH2A from multiple branches of invertebrate evolution^43^ can suggest that mH2A does not have an essential evolutionary-conserved developmental role, but the crucial role of mH2A in some vertebrate species cannot be discarded and is actually supported by our results. Differences in conservation and genomic localization of mH2As among species could be analysed to establish possible correlation with their differences in developmental role effect. Zebrafish mH2A1 shows functional and protein sequence conservation to human and mouse mH2A1.1 splice variant^21^ with 75% protein sequence homology, and further analysis and comparison of splice forms can bring light to observed differences in KO animals among species.

Further work is needed to unveil the mechanisms by which mH2A is affecting gene expression and genome architecture depending on specific cell type, and stage of development. It is attractive to hypothesize that large macrodomain region could have a role forming a signaling platform to recruit histone modifiers, ligands or transcription factors. The use of chimeric proteins with this macrodomain or proteomic analysis of protein complex using *in vivo* models as the one we describe here, could help understand the mH2A regulation network.

## Methods

### Embryo staging and maintenance

Experiments were carried out in accordance with Spanish Institutional Animal Use and Care Committee regulations and with the approval through the Regional Andalusian Government (code A/ES/14/43). Zebrafish (*Danio rerio)* were maintained and raised in a re-circulating water system (Zebtec, Tecniplast) under standard conditions^44^. Zebrafish embryos were obtained from spontaneous spawning and collected out as described in ‘The Zebrafish Book’^45^. Embryos were grown at 28 °C in E3 fresh medium and staged according to the standard morphological features of fish raised at 28.5 °C described by Kimmel and colleagues^26^. Stages were expressed as hours post-fertilization (hpf) at 28.5 °C. Wild type embryos were obtained from AB × TL wild type crosses.

### Antibody, reagents

For western blot analysis we generated specific antibodies against zebrafish mH2A1 and mH2A2 by immunization of rabbits with RMLRFFRRGLPKYRI peptide for mH2A1 and MRYLRTGTHKYRIGM peptide for mH2A2. After bleeding, antibodies were affinity purified (EUROGENTEC; http://www.eurogentec.com).

ChIP-seq data sets were obtained using commercial ChIP grade anti GFP antibody (Abcam ab290).

We also used the following antibodies for immunofluorescence and western blot: H3K27me3 (C36B11), H3K9me3 (Millipore 05-1250), histone H3 (Santa Cruz Biotechnology sc-517576), GFP (Santa Cruz Biotechnology sc-101536). DAPI for nuclear staining (Sigma) and rhodamine phalloidin conjugate (Sigma) for cytoskeleton staining.

### qRT-PCR

RNA was isolated using TRIZOL reagent according to manufacturer’s protocol. First-strand cDNA was primed via oligodT oligonucleotides and RT-PCR was performed with primer sets described in Supplementary Table S12. For quantitative RT-PCR, brilliant SYBR green was used (Biorad).

### Western blot

Dechorioned embryos were lysed using SDS lysis buffer plus protease inhibitors (cOmplete^™^ Protease Inhibitor Cocktail –Roche). Samples were boiled and used for SDS-PAGE and western blot assay with specific antibodies as elsewhere described^45^.

### Chromatin Immunoprecipitation (ChIP)

We used commercial ChIP-validated anti-GFP antibody, and we performed a series of chromatin immunoprecipitations (ChIPs) in biological replicates. We followed published protocol^46^ with small modifications described in the Supplementary Methods Section. Libraries were prepared according to Illumina NEBNext Ultra II DNA Library Prep Kit instructions for DNA sequencing at GENyO genomics facilities.

### RNA-seq experimental procedure

An average number of 350 embryos/isoform per stage where used. RNA was extracted from cell pellet according to the protocol of the RNeasy Plus Micro Kit (Qiagen). The supernatant was passed through a Microsep Advance Centrifugal Device (30 K, Pall) and RNA on the filter was extracted, in order to recover the maximum amount of RNA. The RNA was re-suspended in RNase-free H_2_O from the kit and kept at −80 °C till the RNAseq. RNA was quantified, and quality was checked by Qubit™ RNA HS Assay by the CABIMER genomics facility and libraries were prepared according to Illumina TruSeq Stranded mRNA instructions for RNA sequencing.

### ChIP-seq and RNA-seq data analysis

Both types of sequencing data were generated on the Illumina NextSeq. 500 platform using 4 lanes per sample and 75 bp read length. RNA-seq experiments were run using paired-end sequencing whereas single-end sequencing was used for the ChIP-seq experiments. Quality control of the raw reads was done using FASTQC (v. 0.11.5) separately for each lane of a sample^47^. Identified overrepresented sequences and other adapter and similar technical sequences were identified and subsequently removed by Trimmomatic (v 0.38) in the palindrome mode, based on default alignment detection and scoring parameters^48^. Maximum information quality filtering followed by a minimum average read quality threshold of 25 was used in Trimmomatic for low quality base filtering. Following technical sequence and low-quality base removal, reads that were shorter than 40 bp were filtered out. No lane effects were observed, so reads of a sample in the different lanes were combined for subsequent analysis.

We used four control stages to identify the target genes of an isoform at a time point resulting from the ChIP-seq analysis. First, each sample had an accompanying input DNA to accurately calculate and normalize the enrichment of ChIP DNA targets. Second, we used two wild type (WT) samples, one at each time point (accompanied by their input DNA samples, i.e. 4 samples total) as negative control to detect antibody unspecific binding. Third, we used GFP-only ChIP DNA in two biological replicates at the two time points (accompanied by their input DNA samples, i.e. 8 samples total) as negative control to detect GFP unspecific binding. Finally, we ran a ChIP experiment for a single sample without antibody to detect the background binding noise. For the two isoforms, we ran two biological replicates at the two time points (accompanied by their input DNA samples, i.e. 16 samples total). In total, the ChIP-seq experiment consisted of 29 sequence data files (i.e. samples – ChIP or input DNA), each run on four lanes in single-end mode.

RNA-seq experimental design involved transgenic fish expressing the mH2A1.1 isoform at the two developmental stages where samples with and without yolk were run in duplicates (i.e. 8 samples total). mH2A2 samples at the 75% epiboly stage were run in duplicate and at the 24 hpf stage in duplicate both for GFP positive and GFP negative cell populations, for a total of 6 samples. Each sample was run on four lanes in paired-end mode.

Following trimming and filtering, on average, the ChIP-seq and RNA-seq experiments generated ~18M and ~22M reads per sample, ~71.0 base pair (bp) and ~72.2 bp read length, ~34.6 and ~34.5 average quality score per read, and ~95.9% and ~96.2% of bps above a quality score of 25, respectively. All samples showed high quality and were carried on for subsequent data analysis.

ChIP-seq data analysis was performed by implementing the Bioconductor pipeline: QuasR, mosaics, and ChIPseeker^49^. ‘QuasR’ and ‘mosaics’ were used for read mapping and peak calling, while ‘ChIPseeker’ was used for annotation. GRCz10 (danRer10) was used as the reference genome assembly. We used a bin size of 200 bp, a fragment length of 300 bp, and a False Discovery Rate (FDR) of 0.05 to determine the peaks in ChiP-Seq analysis, whose boundaries were broadened and adjusted using a Hidden Markov Model (HMM) based fitting approach as previously described^49^. The targets for isoforms were identified by subtracting the corresponding WT, GFP, and background peaks after enrichment normalization based on the input DNA were performed. For each isoform/timepoint combination, we categorized the binding regions of the peaks as one of the following based on genome annotation: promoter, UTR, exon, intron, or intergenic. We then calculated the significance of association of the peaks with a genomic region using *regioneR*^50^, which uses a permutation test-based approach making it free from other statistical model assumptions.

In the RNA-seq analysis, transcript quantification was done based on the same reference genome using Salmon (v. 0.8.2) with default parameters^51^. Salmon uses sample-specific models such as correction for GC-content bias that improves the accuracy of transcription abundance estimates. We used Transcripts Per Million (TPM) in Salmon’s output as the normalized relative abundance measure employed in our downstream analysis. Differential gene expression analysis was done using DESeq2^52^, which has been shown to perform well in experimental designs with few replicates^53^. DESeq2 uses a negative binomial model to assess differential expression and employs the Benjamini Hochberg procedure^54^ for multiple hypotheses testing correction. When comparing the transcription abundance between two groups of samples, we used the adjusted p-value cut-off of 0.05 to define statistically significant differential expression.

### Embryo injection and analysis

We obtained mH2A1 and mH2A2 and control morpholinos from Gene Tools (listed in Supplementary Table S12). We generated capped, poly(A) tail mRNA by *in vitro* transcription using the mMessage mMachine Kit (Ambion). We injected fertilized eggs with 100 ng mRNA or 5 ng of morpholino. We staged the embryos as described^26^ and fixed them in 4% (v/v) paraformaldehyde in PBS overnight at 4 °C. We carried out *in situ* hybridization essentially as described^55^.

## Supplementary information

Supplemmentary Methods, figures and figure legends

Video1-GFP-mH2A1

Video2-GFP-mH2A2 6–10 hpf

Video3-GFP-mH2A2 10–24hpf

Supplementary table S1

Supplementary table S2

Supplementary table S3

Supplementary table S4

Supplementary table S5

Supplementary table S6

Supplementary table S7

Supplementary table S8

Supplementary table S9

Supplementary table S10

Supplementary table S11

Supplementary table S12

## Data Availability

The sequencing data from this publication have been deposited to the NCBI Sequence Read Archive (SRA) database (https://www.ncbi.nlm.nih.gov/sra) and assigned the identifier SRP162371.
