# Supplementary material for: Zebrafish macroH2A variants have distinct embryo localization and function
Source: Sci Rep. 2019 Jun 14;9:8632. doi: 10.1038/s41598-019-45058-6 (PMC6570772; doi:10.1038/s41598-019-45058-6)

Supplementary Information

Zebrafish macroH2A variants have distinct embryo localization and function

Gonzalez-Munoz, E.*,[1,2,3], Arboleda-Estudillo, Y.[4], Chanumolu, S.K. [5], Otu, H.H. [5], Cibelli, J.B*,[4,6].

1 LARCEL, Andalusian Laboratory of Cell Reprogramming (LARCel), Andalusian Center for Nanomedicine and Biotechnology-BIONAND, 29590 Málaga, Spain. egonmu@uma.es
2 Department of Cell Biology, Genetics and Physiology, University of Málaga, 29071 Málaga, Spain.
3 Networking Research Center on Bioengineering, Biomaterials and Nanomedicine, (CIBER-BBN), 29071 Málaga, Spain.
4 LARCEL, Andalusian Laboratory of Cell Reprogramming (LARCel), Andalusian Center for Nanomedicine and Biotechnology-BIONAND, 29590 Málaga
5 Department of Electrical and Computer Engineering, University of Nebraska-Lincoln
6 Department of Animal Science and Large Animal Clinical Sciences Department, Michigan State University, East Lansing, MI 48824, USA.

(*) Corresponding authors: egonmu@uma.es; cibelli@msu.edu.

Supplementary Figure legends

Supplementary Figure S1. Identification of mH2A promoter regions.
We PCR amplified 5 kbp product from wild-type AB zebrafish genomic DNA of mH2A1 (**A**) and mH2A2 (**B**) genes, upstream from the start of the open reading frame of each gene. We selected and amplified specific regions with promoter-like properties (region locations are indicated in each table relative to ORF starting nucleotide triplet, ATG). These PCR fragments were cloned into a modified Tol2 expression vector containing the EGFP reporter transgene and where injected into 1–2 cell stage AB embryos, for GFP expression detection at 75% epiboly and 24 hpf stages. Table shows in red font the selected candidate promoter regions matching endogenous expression pattern at these stages.

Supplementary Figure S2. Under a constitutive promoter, mH2A1.1 fusion protein is expressed throughout the zf embryo but only in the YSL when expressed under the endogenous promoter.
In vivo confocal imaging of transgenic zf embryos expressing EF1:GFP-mH2A1.1 (upper panel) or mH2A1:GFP-mH2A1.1 (lower panel). Figure shows two representative series of merged brightfield-GFP snapshots at different developmental stages during live capturing. This result shows that fusion protein GFP-mH2A1.1 localization on YSL is due its promoter regulated expression and not due to further protein processing or fusion protein mislocalization. (Assay conducted in triplicate with n=10-15 embryos/ isoform).

Supplementary Figure S3. Zebrafish fusion proteins GFP-mH2A1.1 and GFP-mH2A2 can colocalize with heterochromatin markers.
A. Transgenic zf embryos expressing ubiquitously GFP-mH2A1.1 fusion protein EF1:GFP-mH2A1.1 at 60% epiboly stage were analysed using immunohistochemistry to detect heterochromatin markers trimethyl Histone3 lysine K27 (H3K27me3). DAPI was used for nucleus labelling. Confocal microscope image projection of 60% epiboly stage transgenic embryo shows partial embryo body colocalization. Labelling assay was conducted in triplicate with n= 40-50 embryos/assay.

Supplementary Figure S4. Zebrafish ubiquitously expressed fusion protein mH2A1-GFP colocalize with mitotic nucleus.
Transgenic zf embryos expressing ubiquitously GFP:mH2A1.1 75% epiboly stage were analysed using immunohistochemistry to detect mitotic nuclei

phosphohistone H3 (pHH3) positive. DAPI was used for nucleus labelling. mH2A1.1 colocalize with mitotic PHH3 positive cells, indicating no cell proliferation blocking effect in these cells. Labelling assay was conducted in triplicate with n= 40-50 embryos/assay.

Supplementary Figure S5: mH2A2 and mH2A1 are required for proper zebrafish embryo development.

A. Lateral and apical view of fixed 24 hpf embryos after downregulation of mH2A isoforms using specific morpholino antisense oligos (MO-mH2A1, MO-mH2A2) or non-specific control MO (MO-CTRL) show alteration in 4th ventricle formation and optic tectum in lateral views (arrows), and head rostral structures in apical view (arrow, arrowhead and hollow arrowhead point mensencephalon, cerebellum and telencephalon respectively *[46, 47]) .*

Assay was performed in triplicate. Control and specific MO injected embryos (n=85-100/ each) were analyzed in these experiments. 90% (MO-mH2A1) and 92% (MO-mH2A2) of injected embryos showed uniform described phenotype. Control phenotype was recovered by injection of embryos with mH2A1 or mH2A2 morpholino in combination with mH2A1.1 or mH2A2 mRNA (80 and 85% of recovery).

B. Characterization of morphants and control embryos at the 18-somites stage. Lateral view of in situ RNA hybridization shows alteration of the midbrain-hindbrain-boundary (MHB) using MHB marker pax2a.

Rescues were generated by injection of embryos with mH2A1.1 or mH2A2 morpholino in combination with mH2A1 or mH2A2 mRNA.

Experiment was performed in triplicate. Control and specific MO injected embryos (n=120/ each) were analyzed in these experiments with 90% of them, showing uniform described phenotype.

* In all experiments we co-injected a morpholino against p53 (p53 MO) to mitigate the nonspecific dose-dependent neural toxicity widely reported [48]

Supplementary Figure S6:

Sample Chip-seq peaks and areas around them at the two time points using the Integrative Genomics Viewer (IGV): A) for the two isoforms B) for the negative controls. The results show high read counts in the binding regions relative to the areas around them for the two isoforms (A) and insignificant read counts for the negative controls (B). Peak profiles are shown for both replicates (blue: replicate 1, pink: replicate 2) following correction with input DNA.

Supplementary Figure S7: Isoform specific target DNA correlation with relative RNA expression profile.

Correlation of mH2A1 and mH2A2 genome occupancy with the relative transcriptional level (Fold change, FC) of the significantly differentially expressed target genes between samples: **A.** mH2A1.1 at 75% epiboly and 24 hpf stage **B**. mH2A2 at 75% epiboly and 24 hpf stage. **C.** mH2A1.1 and mH2A2 at 75 epiboly stage and **D.** mH2A1.1 and mH2A2 at 24 hpf stage.
Venn diagrams are identical to Figure 6B and graphs show correlation (enrichment of the specific isoform in a regulatory region of a gene with the relative expression level of the gene). Genes were grouped into upregulated (FC>1.5) or downregulated (FC<-1.5) and further grouped into eight categories according to their FC range (Overexpressed: 1 (FC 1-2); 2 (FC 2-4); 3 (FC 4-10); 4 (FC >10) or decreased: -1 (FC 1-2); -2 (FC 2-4); -3 (FC 4-10); -4 (FC >10)).

Supplementary Figure S8: Average effect of isoform binding in gene expression level indicates different trend on repressing or activating effect but not a broad inhibitory effect.

Percentage of genes in each of the four z-score intervals. Z-scores are calculated based on the log-transformed “transcripts per million” (TPM) values. Positive z-scores imply high expression, negative z-scores imply low expression. Blue bars represent results for all the genes and orange bars represents results for only the genes targeted by the isoform. For each of the blue-orange bar pair, the significance of the difference is assessed using Fisher’s Exact Test. For example, 4.73% of all the genes in I124 have a z-score greater than 1.5 while only 1.27% of the mH2A1’s target genes have a z-score 1.5. This difference is significant and the target genes for mH2A1 at 24hpf are depleted in this high expression interval. I1E: mH2A1-75% epiboly, I124: mH2A1-24hpf, I2E: mH2A2-75% epiboly, I224: mH2A2-24hpf, **: p< 0.05, ***: p<0.001.

Supplementary Figure S9: Transcript variant contribution to mH2A1 expression.
Quantitative RT-PCR was performed for total mH2A1, and for h2afy-201 and h2afy-202 5’UTR specific transcripts. Data show contribution of both variants to total mH2A1. mRNA with h2afy-202 showing the highest contribution. Mean values (n=3) ± SEM are plotted. Values indicate relative expression of the specific gene normalized to GAPDH/TUBULIN

Supplementary Figure S10: Full-length blots of figure 7A.

Supplementary Video 1: Confocal time lapse imaging of mH2A1:GFP-mH2A1.1 transgenic zf embryos development. Image acquisition started at 6 hpf and finished at 28 hpf.

Supplementary Video 2: Confocal time lapse imaging of mH2A2:GFP-mH2A2 transgenic zf embryos development. Image acquisition started at 6 hpf and finished at 10 hpf

Supplementary Video 3: Confocal time lapse imaging of mH2A2:GFP-mH2A2 transgenic zf embryos development. Image acquisition started at 10 hpf and finished at 22 hpf

SUPPLEMENTARY METHODS

Antibody, reagents

For western blot analysis we generated specific antibodies against zebrafish mH2A1 and mH2A2 by immunization of rabbits with RMLRFFRRGLPKYRI peptide for mH2A1 and MRYLRTGTHKYRIGM peptide for mH2A2. After bleeding, antibodies were affinity purified (EUROGENTEC; http://www.eurogentec.com).

ChIP-seq data sets were obtained using commercial ChIP grade anti GFP antibody (Ab 290 AbCam). We also used the following primary antibodies for immunofluorescence and western blot: H3K27me3, H3K9me3 (cell signaling), H3 (Santa Cruz Biotechnology), pHistone-H3-Ser10 (1/600, Santa Cruz). We used HOECHST 33342 for nuclear staining (Sigma-Aldrich) and rhodamine phalloidin conjugate (Sigma-Aldrich) for cytoskeleton staining.

Vectors and constructs

We generated pTOL2 constructs using PT2KXIGDin vector (kind gift of Marion Delous)[49]. mH2A1 and mH2A2 promoter regions or Xenopus EF1α enhancer /promoter region (for ubiquitous expression of downstream cassette) were cloned using XhoI/SalI restriction sites and GFP-mH2A1 and GFP-mH2A2 fusion proteins using BamHI/ClaI restriction sites. mH2A1.1 and mH2A2 coding region cDNA sequences where cloned from zebrafish total mRNA using PCR oligonucleotides listed in supplementary table S12. After sequencing cloned transgenes we confirmed cDNA sequences correspond to NM_001040361 (mentioned highly homolog to mammal mH2A1.1 and thus labeled as mH2A1.1) and NM_001025502 (mH2A2) NCBI reference sequences respectively. RT-PCR analysis using UTR specific primers for mH2A1 spliced variants (h2afy-201 and h2afy-202 transcripts) show contribution of both variants to total mH2A1 mRNA with h2afy-202 showing the highest contribution (Figure S9).

Tol2-transposase-mediated transgenesis was used to produce stable transgenic lines.

Sequences for promoter region cloning, GFP-fusion proteins cloning, and RT-PCR oligonucleotides used are listed in supplementary table S12.

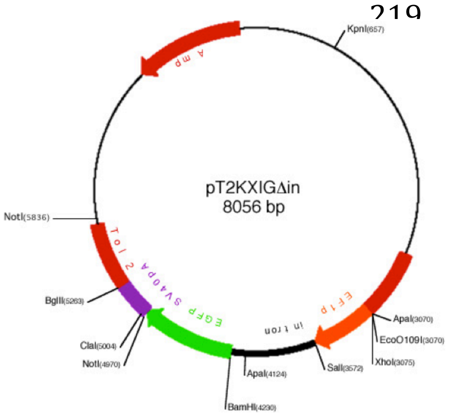

pGEM-T Easy vector was used to clone partial sequences of the mH2A1.1 and mH2A2 cDNAs for antisense RNA probes synthesis using oligonucleotides listed in supplementary table S12.

Micro-injections for transgenic zebrafish lines generation

For genome integration and generating stable transgenic fish lines, Tg(EF1:GFP-mH2A1.1), Tg(mH2A1:GFP-mH2A1.1), Tg(mH2A2:GFP-mH2A2), one-cell-stage wild type embryos were injected with 25ng/µL *Transposase (TP)* mRNA + 25ng/µL pTOL2 constructs based on the Tol2 vector system [51], using a motorized Eppendorf Patchman NP2 and Eppendorf FemtoJet. A *pTol2*-transfer vector containing an insert encoding the transposase protein was linearized with the NotI-HF restriction enzyme (New England BioLabs). *Tol2 Transposase* RNA was synthesized with the SP6 mMessage mMachine kit (Ambion) following the kit instructions. Injected embryos (F0) were screened for expression of GFP under a fluorescent stereo microscope. Embryos showing specific expression of GFP in the nuclei were raised to adulthood and crossed with wild type fish in order to identify founder fish with GFP positive offspring. From these, stable transgenic lines were established.

Genotyping of the transgenic lines

All injected fish (F0) were raised to sexual maturity and crossed in pairs to identify germline chimeras. The genotype of progeny embryos of F0 Tg(mH2A1:GFP-mH2A1.1), and Tg(mH2A2: GFP-mH2A2) microinjected fish was identified by PCR of genomic DNA extracted from tail fin using specific primers. F1 progeny of each germline F0 fish were then raised to adulthood and identified by epifluorescence using a Leica M165FC fluorescence stereo

microscope equipped with a 1.0X PlanApo objective, a MDG base, and a Hamamatsu Orca R^2 C10600 camera.

qRT-PCR.

RNA was isolated using TRIZOL reagent according to manufacturer´s protocol. First-strand cDNA was primed via oligodT oligonucleotides and quantitative PCR was performed with primer sets described in the supplementary table S12 and brilliant SYBR green (Biorad).

Western blot

Dechorionated embryos were lysed using SDS lysis buffer plus protease inhibitors (cOmplete™ Protease Inhibitor Cocktail –Roche). Samples were boiled and used for SDS-PAGE and western blot assay with specific antibodies as elsewhere described [36]**.** (Briefly: (0.1% SDS, 50 mM HEPES pH 7.4, 10 mM EDTA, 150 mM NaCl, 10 mM sodium pyrophosphate, 100 mM sodium fluoride, 1 mM sodium vanadate and a tablet of C-complete (Roche) protease inhibitor cocktail. After centrifugation at 12,000g for 15 min, 100ug of protein supernatant was resuspended in Laemmli SDS-DTT sample buffer for western blot analysis using the specific antibodies.

For western blot analysis of nuclear zebrafish proteins, we disaggregated whole embryos in hypotonic lysis buffer (10mM Tris, 85mM KCl, 0.5% (w/v) Triton X100, 1mM PMSF) and collected nuclei by mild centrifugation.

Chromatin Immunoprecipitation (ChIP). We followed the protocol published by Lindeman and colleagues [37] with small modifications, briefly, 500 embryos at 75% epiboly stage and 24 hpf from wild type, *Tg(mH2A1:GFP-mH2A1.1)* *Tg(mH2A2:GFP-mH2A2)* and *Tg(H2A:GFP)* [52] were disaggregated in PBS (Gibco) containing 20 mM Na-butyrate (Sigma-Aldrich) and Protease Inhibitor (1/100, cOmplete Mini, Roche) by mechanically passing them through a 23G needle (BD Microlance). One percent of 36,5-38% Formaldehyde solution (Sigma) was used to cross-link the DNA of the cells and 0,125 M of glycine (Sigma) was used to quench excess of formaldehyde. After centrifugation at 4˚C, PBS + protease inhibitor was used to wash the pellets. The samples resuspended in lysis buffer were sonicated on ice 6 times for 10 seconds each with a Branson Digital Sonifier. After centrifugation at 4˚C, the absorbance of the chromatin contained in the supernatant was measured at 260 nm with a Nanodrop 2000 (Thermo Scientific). The protein concentration was measured following the Pierce BCA Protein Assay Kit manufacture protocol (Thermo Scientific).

Dynabeads Protein G (Novex Life Technologies) resuspended in Ripa buffer and incubated at 4˚C during 2 hours with 0,05-0,1 ng/µL of the Anti-GFP antibody ChIP Grade (Ab 290 AbCam) were used to immunoprecipitate the chromatin. Briefly, after removing the buffer with a magnetic rack to trap the beads, the chromatin was added to the beads containing the antibody and

incubated at 4˚C overnight. The magnetic rack was used for the next washes with Ripa buffer and TE buffer. ChIP elution buffer was used to reverse the formaldehyde-induced cross-links by incubating the samples on a thermoshaker (Grant Bio™ PMHT) at 1300 rpm at 68˚C for 2 hours. The input chromatin samples were treated with proteinase K (2 mg/ml, Roche) and incubated on the thermoshaker at 1300 rpm at 68˚C during 15 min. The purification of immunoprecipitated and input DNA DNA was done by phenol:chloroform:isoamylalcohol (Invitrogen) extraction.

FACS sorting of GFP positive cells from zebrafish larvae and total RNA isolation for RNA seq.

A pool of about 600 larvae was dechorionated and deyolked as described by [53].

Total RNA for RNA-seq was obtained from dechorionated *Tg(mH2A:-GFP-mH2A1.1)* and *Tg(mH2A2:GFP-mH2A2)* embryos at 75% epiboly stage and 24 hpf with and without previous deyolking, following the protocol of the RNeasy Plus Mini Kit (Qiagen). The RNA was re-suspended in DEPC-treated H_2O from the kit and kept at -80˚C. RNA for RNA-seq from GFP positive cells from *Tg(mH2A2:GFP-mH2A2)* embryos at 75% epiboly and 24 hpf stages was obtained using fluorescence-activated cell sorting (FACS) on a BD FACSAria Cell Sorter (BD BioSciences) using wild type embryos as GFP negative control. Briefly, the embryos were dechorionated with pronase (2 mg/mL, Roche) and deyolked as described above. Embryos were mechanically disaggregated in Hank´s Balanced Salt Solution (HDSS, Gibco) without Ca^{2+} and Mg^{2+} containing 10% FBS, by passing the embryos through a cell strainer with 40 µm pore size (Falcon) with the help of a 1mL syringe plunger. From the disaggregation of the embryos all the steps were carried out at 4˚C. Single cells were washed 2 times, centrifuged and re-suspended again in HBSS + FBS for FACS sorting. Cells were sorted at 4˚C and collected in 15 mL falcon tubes containing 10% RNAlater (Thermofisher).

After cell sorting, the cells were pelleted, and RNA was extracted from the pellet according to the protocol of the RNeasy Plus Micro Kit (Qiagen). The supernatant was passed through a Microsep Advance Centrifugal Device (30K, Pall) and RNA on the filter was extracted, in order to recover the maximum amount of RNA. The RNA was re-suspended in RNase-free H_2O from the kit and kept at -80˚C till the RNAseq.

Morpholino oligonucleotide knockdowns

Morpholino (MO) antisense oligonucleotide were synthesized and obtained by Gene Tools, Inc. MOs were designed to reduce the mH2A1 or mH2A2 expression (listed in supplementary table S12).

A MO control was used in order to account for potential non-target MO toxicity.

MO stock solutions (1 milliMolar) were prepared by re-suspending lyophilized MOs in molecular biology grade water, and stocks were stored at -20˚C. To reduce mH2A1 or mH2A2 expression, we co-injected one-cell-stage wild type embryos with 0,5 nL of 0.25 mM *mH2A1, mH2A2 or CTRL* (0,125 mmoles) MOs + 0.5 mM (0,25 mmoles) *p53* MO to mitigate the neural toxicity [48] and 0,125 mmoles of MO + 200 pg GFP-mH2A isoform mRNA for the rescue. Phenotypes were examined by using a Leica M165FC fluorescence stereo microscope equipped with a 1.0X PlanApo objective, an MDG base and a digital camera DFC495. Images were assembled in Photoshop.

Wholemount in situ hybridization

Whole-mount *in situ* hybridization was performed as previously described by Thisse & Thisse [45] with minor modifications. Antisense RNA probes were generated against *goosecoid* (*gsc*, from Heisenberg, C.P lab), *green fluorescent protein* (*gfp*, kind gift of Skarmeta, J.L lab), *pax2a* (kind gift of Brand, M. lab) and *engrailed2* (*en2*, kind gift of Oates, A.C. lab).Probes and wild-type expression patterns were as previously described for: *gsc* [54] *pax2a* [55], and *eng2* [56, 57]. mH2A1 and mH2A2 probes were synthesized from ScaI digested pGEM-T Easy vectors described above. RNA antisense probes were synthesized using a DIG RNA labeling KIT (Roche) and the SP6/T7 mMessage mMachine kit (Ambion) following manufacturer's instructions. DIG labeled probes were purified with Lithium Chloride. Embryos were fixed overnight with 4% paraformaldehyde (Sigma) at 4˚C at 75% epiboly stage (8 hpf) and at 24 hpf. To visualize the probes Anti-Digoxigenin-Alkaline Phosphatase (AP) Fab Fragments and BM Purple AP Substrate (Roche) were used. The AP enzyme was used to convert BM Purple into a precipitating violet staining. Whole-mount *in situ* hybridization at 75% epiboly stage and 24 hpf were photographed using a Leica M165FC fluorescence stereo microscope equipped with a 1.0X PlanApo objective, a digital camera DFC495. Images were assembled in Photoshop.

Whole-Mount Antibody Staining

Wild type, *Tg(mH2A:-GFP-mH2A1.1)* and *Tg(mH2A2:GFP-mH2A2)* fish embryos were incubated at 28˚C and fixed at 75% epiboly and 24 hpf in 4% paraformaldehyde overnight at 4˚C. Dechorionated embryos were blocked with PBS-0.3%Triton-5%serum for 5 hours at 4ºC and incubated overnight with pHistone-H3-Ser10 (1/600, Santa Cruz), Tri-Methyl-Histone H3-Lys27 (1/300, Cell Signaling Technology) and Trimethyl Histone H3-Lys 9 (1/200, Millipore) primary antibodies. The primary antibodies were visualized using Alexa fluor 594 anti-Mouse (1/300, Invitrogen) for Histone H3K9 and Alexa fluor 594 anti-Rabbit (1/300, Invitrogen) for pHistone H3 Ser10 and Histone H3K27. Hoechst 33342 (Sigma-Aldrich) was used to stain the nuclei.

Confocal Microscopy

In vivo images and images from stained samples of transgenic embryos were obtained with an inverted Leica SP5 confocal microscope equipped with a Plan Apo 25X (0.95 NA water-immersion 2.4mm WD) and Plan Apo 10X (0.4 NA dry) lens with 405 nm, 488 nm and 594 nm laser lines. When referring to maximum intensity projection we used the visualization technique supported by Leica SP5 confocal software, that takes 3D data (in our case a Z-stack of confocal microscope images) and turns it into a single 2D image. The projection takes the brightest pixel (voxel) in each layer and displays that pixel intensity value in the final 2D image.
In vivo images were taken in lateral regions of the gastrula at 20 min time intervals from shield to 24 hpf stages. Images were assembled in Photoshop, recorded as TIFF files and analyzed with the image processing program Image J (Fiji).

Supplementary Figure S1

A

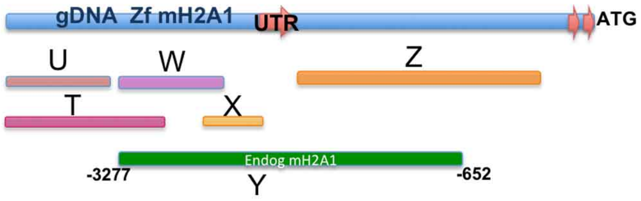

NAME	3´ position	5´ position	GFP+ 75% Epib	GFP+ 24hpf
Z	-140	-1704	+	+
Y	-652	-3277	++++	+++
X	-1841	-3306	-	-
W	-2182	3277	-	-
U	-3277	-4357	-	-
T	-2275	-4357	-	-

B

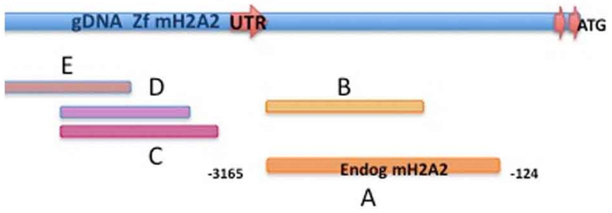

NAME	3´ position	5´ position	GFP+ 75% Epib	GFP+ 24hpf
A	-124	-3165	+	+++++
B	-1408	-3165	-	+
C	-4007	-6118	-	-
D	-4796	-6118	-	-
E	-5314	-6383	-	-

Supplementary Figure S2

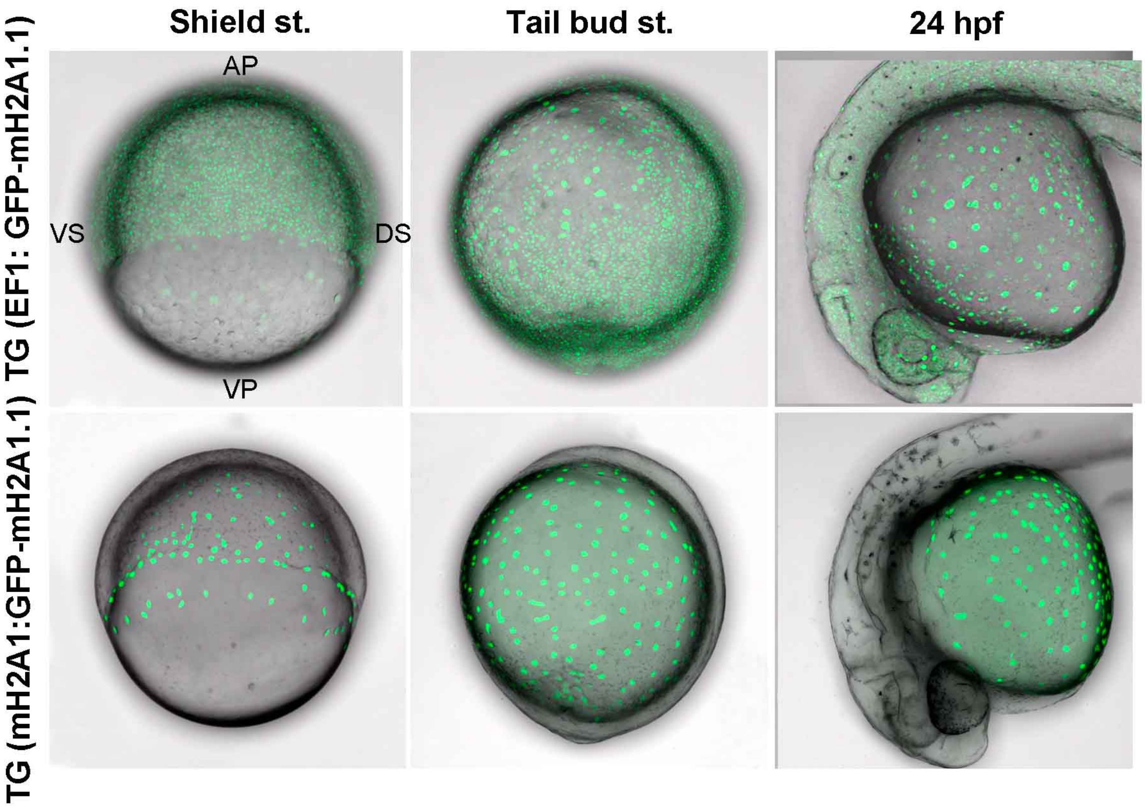

Supplementary Figure S3

A

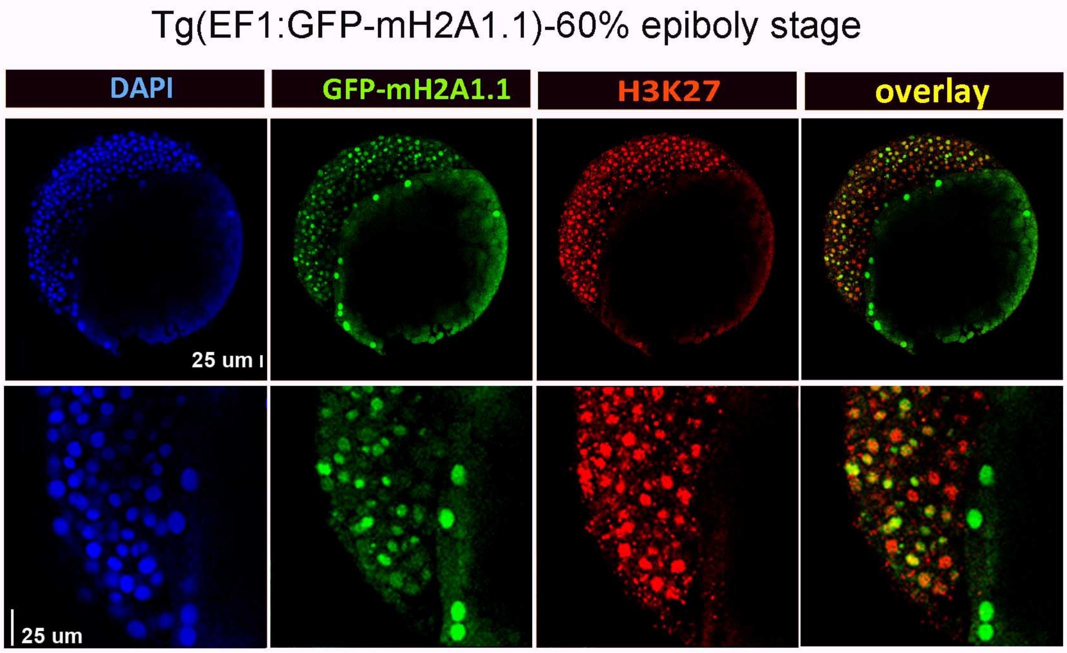

Supplementary Figure S4

Tg(EF1:GFP-mH2A1.1) - 75% epiboly stage

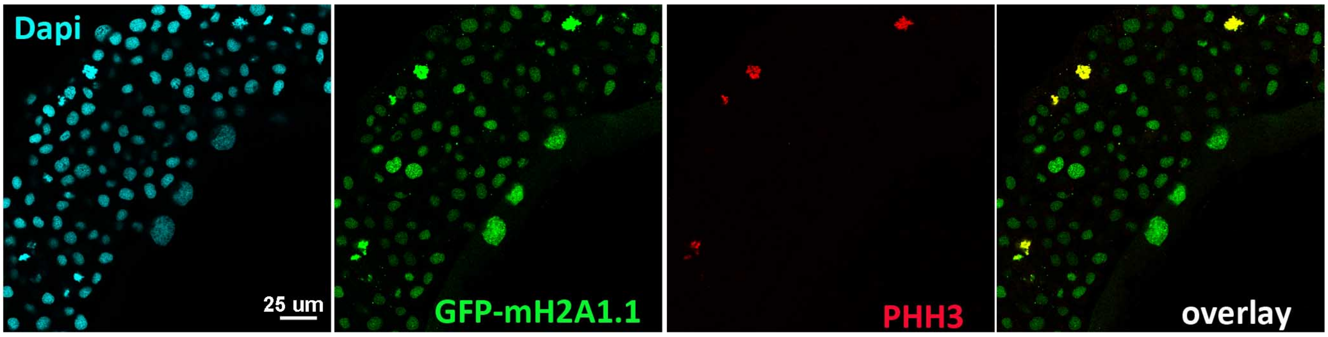

Supplementary Figure S5

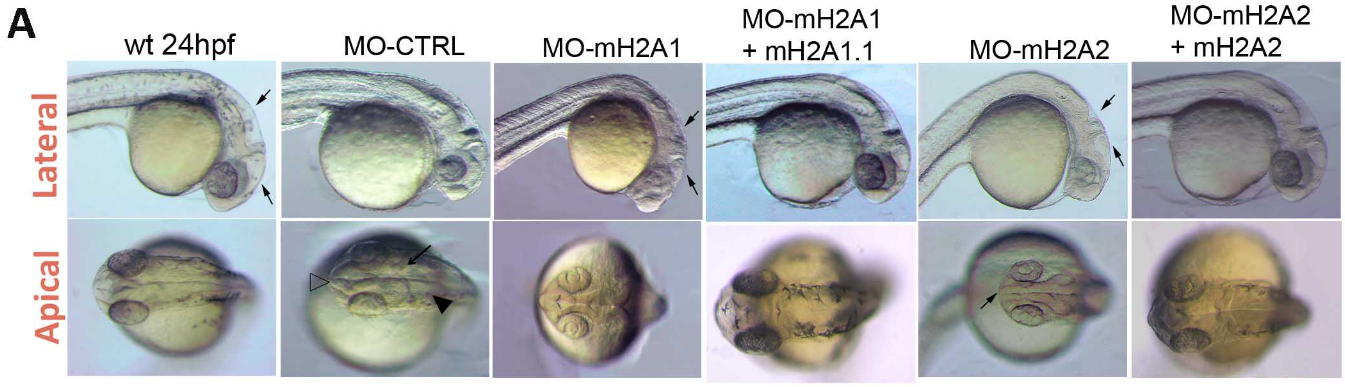

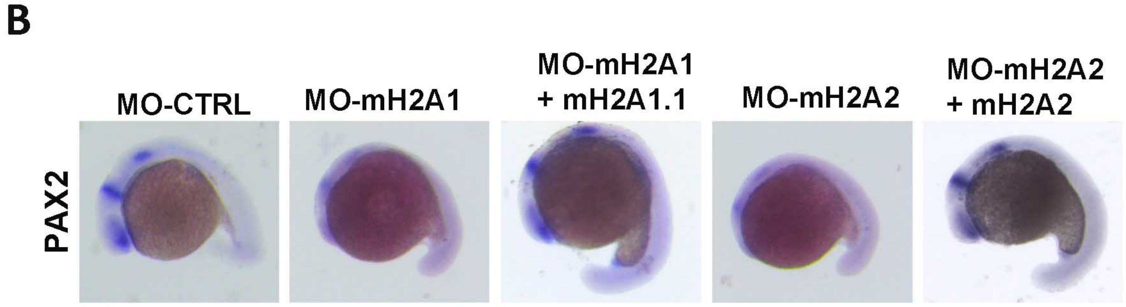

Supplementary Figure S6

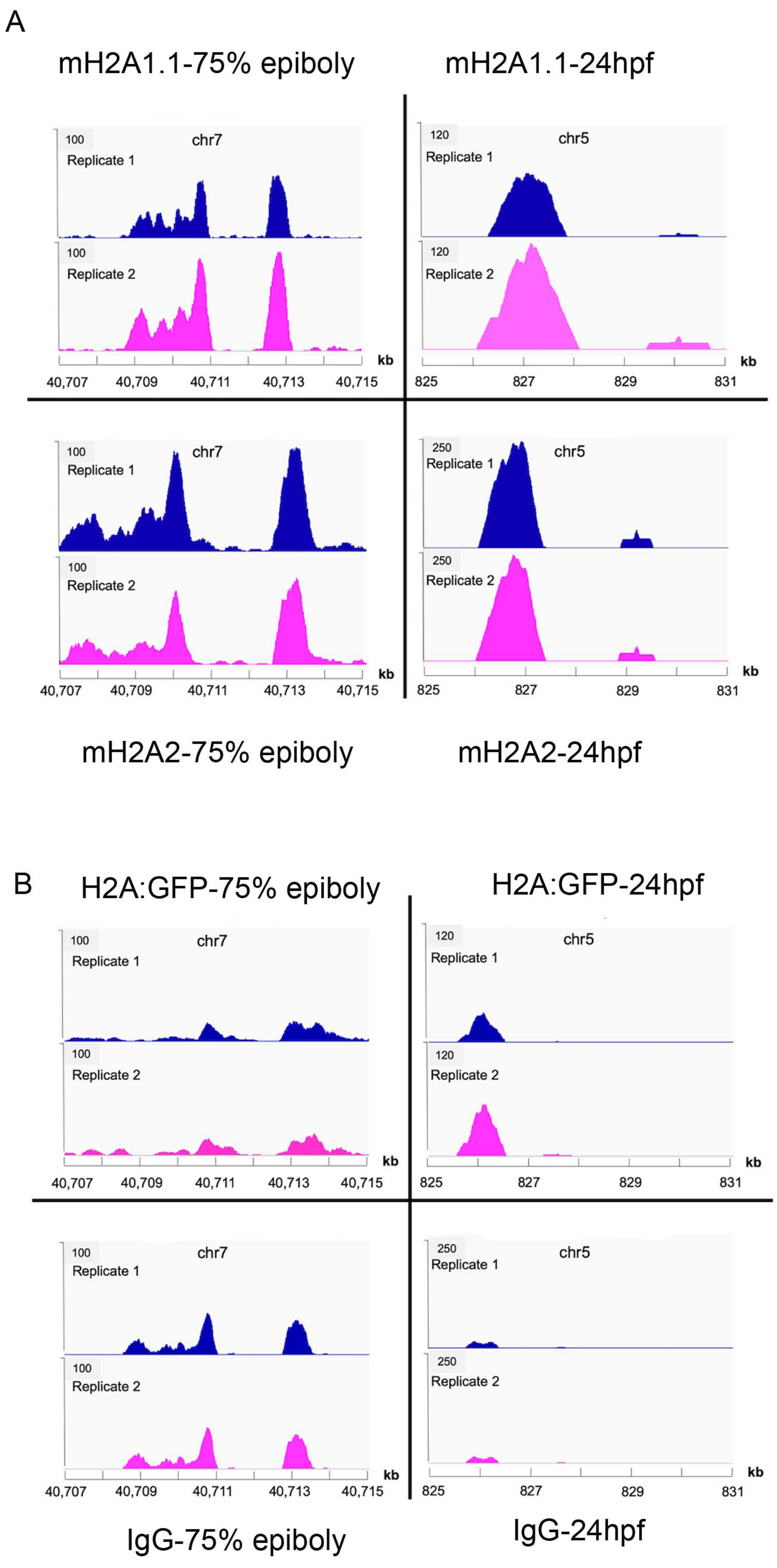

Supplemetary Figure S7

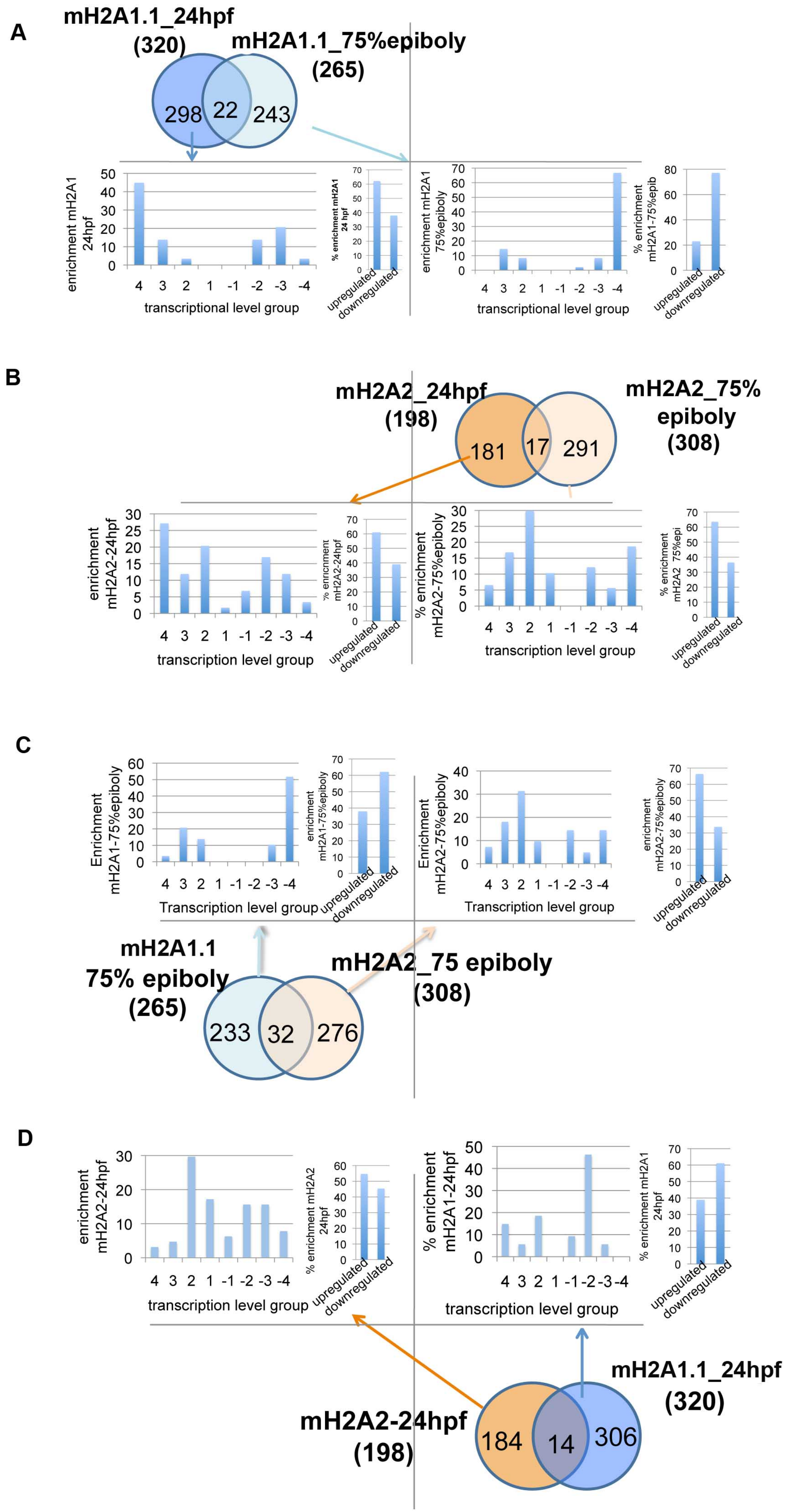

Supplementary Figure S8

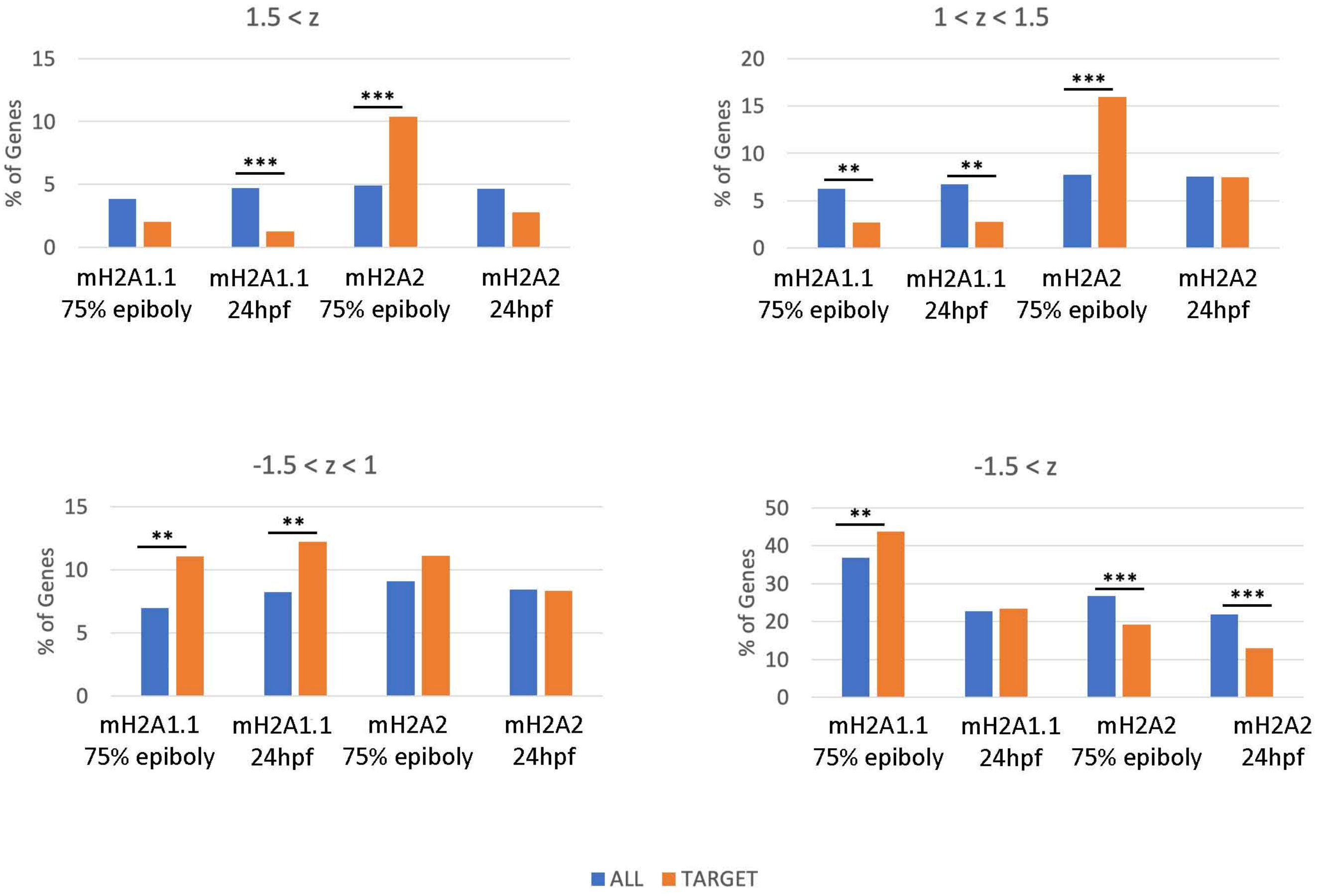

Supplementary Figure S9

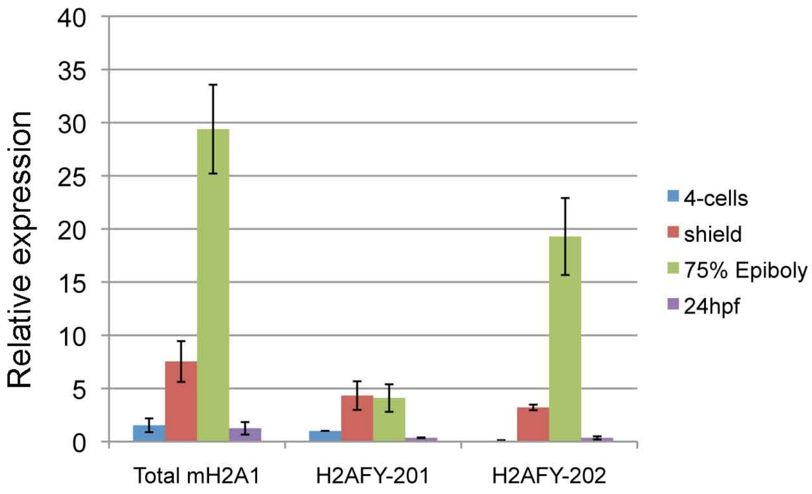

Supplementary Figure S9: Full-length blots of Figure 7A

MO mH2A1: ggatgtccagtcgtggagggaagaa

MO mH2A2: ggatgtcagccagaggaggaaagaa

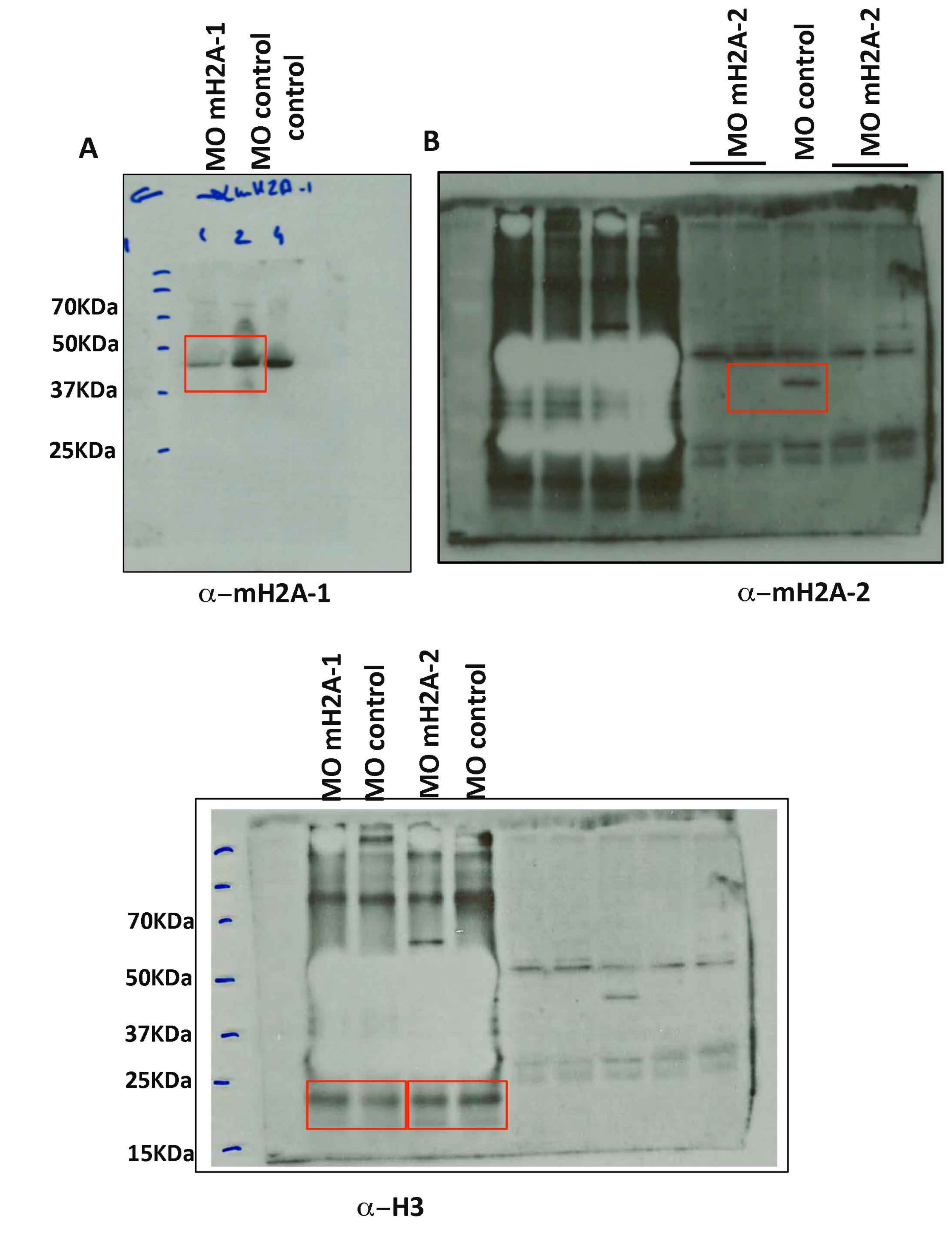

Supplement: Supplementary file 1 — Supplemmentary Methods, figures and figure legends [file 41598_2019_45058_MOESM1_ESM.pdf]
